# Recent Progress in Fabrication and Applications of Superhydrophobic Coating on Cellulose-Based Substrates

**DOI:** 10.3390/ma9030124

**Published:** 2016-02-25

**Authors:** Hui Liu, Shou-Wei Gao, Jing-Sheng Cai, Cheng-Lin He, Jia-Jun Mao, Tian-Xue Zhu, Zhong Chen, Jian-Ying Huang, Kai Meng, Ke-Qin Zhang, Salem S. Al-Deyab, Yue-Kun Lai

**Affiliations:** 1 National Engineering Laboratory for Modern Silk, College of Textile and Clothing Engineering, Soochow University, Suzhou 215123, China; 20154215029@suda.edu.cn (H.L.); 1215402005@suda.edu.cn (S.-W.G.); 2155215013@suda.edu.cn (J.-S.C.); 20155215023@suda.edu.cn (C.-L.H.); 20155215002@suda.edu.cn (J.-J.M.); 20154215018@suda.edu.cn (T.-X.Z.); mk2009@suda.edu.cn (K.M.); kqzhang@suda.edu.cn (K.-Q.Z.); 2 School of Materials Science and Engineering, Nanyang Technological University, 50 Nanyang Avenue, Singapore 639798; aszchen@ntu.edu.sg; 3 Research Center of Cooperative Innovation for Functional Organic/Polymer Material Micro/Nanofabrication, Soochow University, Suzhou 215123, China; 4 Department of Chemistry, Petrochemical Research Chair, College of Science, King Saud University, Riyadh 11451, Saudi Arabia; ssdeyab@ksu.edu.sa

**Keywords:** superhydrophobic cellulose-based materials, wettability, self-cleaning, oil-water separation, wetting pattern

## Abstract

Multifuntional fabrics with special wettability have attracted a lot of interest in both fundamental research and industry applications over the last two decades. In this review, recent progress of various kinds of approaches and strategies to construct super-antiwetting coating on cellulose-based substrates (fabrics and paper) has been discussed in detail. We focus on the significant applications related to artificial superhydrophobic fabrics with special wettability and controllable adhesion, e.g., oil-water separation, self-cleaning, asymmetric/anisotropic wetting for microfluidic manipulation, air/liquid directional gating, and micro-template for patterning. In addition to the anti-wetting properties and promising applications, particular attention is paid to coating durability and other incorporated functionalities, e.g., air permeability, UV-shielding, photocatalytic self-cleaning, self-healing and patterned antiwetting properties. Finally, the existing difficulties and future prospects of this traditional and developing field are briefly proposed and discussed.

## 1. Introduction

**Figure 1:**
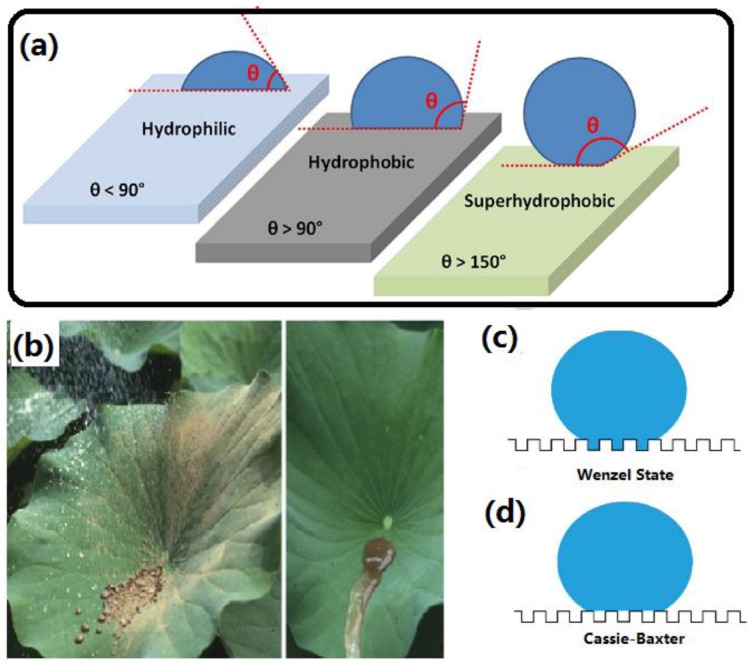
(**a**) The schematic illustration of wetting states of surfaces with different contact angles (Reprinted from Reference [5] with permission); (**b**) Lotus leaf with typical self-cleaning effect; (**c**) Wenzel wetting state; (**d**) Cassie-Baxter wetting state.

In general, the wettability of a surface can be characterized by the contact angle (CA). Multifuntional surfaces with special wettability have attracted a lot of interest in both fundamental research and industry applications over the last two decades [1,2,3,4,5,6,7,8,9,10,11,12,13,14,15]. The surface with a water droplet CA less than 90° and above 90° is commonly defined as hydrophilic and hydrophobic respectively, as demonstrates in Figure 1a. Recently, specific superhydrophobic surfaces with a CA larger than 150° have attracted significant attention because of their unique super-antiwetting, self-cleaning properties and their potential for practical applications. It is well known that numerous creatures and plants exist in nature with amazing superhydrophobic surfaces. For example, the lotus effect [16,17,18], as shown in Figure 1b, describes the excellent super-antiwetting and self-cleaning ability of lotus leaf surfaces. The rainwater can smoothly roll off the lotus leaves instead of sticking to the surface. These special characteristics are attributed to the combination of a waxy layer with low surface energy and dual-scale rough structured surface with protrusions on lotus leaves. If these two key features of lotus leaves can be extended to other substrates, it can be useful and helpful to construct artificial super-antiwetting surfaces for numerous practical applications. Despite the excellent properties of cotton fibers, one of the most abundant and widely used natural materials in our world, some inherent features such as being hydrophilic, poor resistance to UV light (color yellowing), impotent antimicrobial activity, have confined their wider applications, especially in some high-tech fields for self-cleaning, medicine, personal healthcare, and flexible multifunctional textile. Therefore, the value-added cotton fabrics by post-functionalizationhave attracted considerable academic and industrial attention, not only due to their potential utilization in thermal, physical, and biological protection, but also to meet the fast evolving demand from consumers for advanced multifunctional cloths.

Generally, the wetting states of water droplets on solid surface can be classified into two categories, namely Cassie-Baxter state and Wenzel state [19,20,21], in which the water either sits upon the surface protrusions or penetrates into the surface porosity, respectively, as shown in Figure 1c,d. The superhydrophobicity can be usually explained by the Cassie-Baxter model [20]. In this model, a large amount of air is trapped in the microgrooves of a rough surface and water droplets rest on a composite surface comprising air and the tops of micro-protrusions. The importance of the fractal dimensions of the rough surfaces is well recognized and many approaches have been based on the fractal contribution. Suitable roughness in combination with low surface energy has been required to construct artificial superhydrophobic surfaces. Therefore, altering one of these two factors or both will change the surface wettability state.

Surface engineering of cellulose-based substrates with desired functionalities can be achieved by a considerable number of physical and chemical techniques ranging from traditional treatments to multifunctional approaches. Superhydrophobic cellulose-based materials, in fact, offer a challenging platform for functional modifications in order to meet rigorous requirements for a variety of practical applications. This article reviews recent advances involving surface engineering of physical structure and chemical component on cellulose-based substrates, especially for fabric and paper, using wet (dip-coating, wet chemical etching, chemical bath deposition, electro-assistant deposition, and spray-coating) or dry (chemical vapour deposition, and plasma processing techniques) processe. The corresponding properties (robustness, durability, breathability, and self-healing) are discussed too. The authors will also present how the controlled wettability is integrated into traditional cellulose-based materials to improve their super-antiwetting performances and to extend their practical applications by developing new functionalities (oil-water separation, self-cleaning and asymmetric/anisotropic wetting). Finally, the authors give a brief summary and outlook on the fabrication and applications of superhydrophobic cellulose-based surfaces and the emerging development of innovative production techniques used to modify the surface materials and to improve the product quality.

## 2. Construction of Superhydrophobic Coating on Cellulose-Based Substrates 

The construction of surfaces with special wettability is inspired by a wide variety biological creatures and plants, such as lotus leaf, water strider, geckos, butterfly wings, shark skin, fish scale [22,23,24,25,26,27,28]. The combination of micro-nano dual-scale hierarchical structures (surface roughness) and chemical components on biological surfaces is verified to be a vital factor for the realization of superwettability properties. Bio-inspired by these findings, many techniques and rational strategies have been applied to construct robust superhydrophobic and self-cleaning fabrics by mimicking the surface topography of biological systems [29,30,31,32,33].

In general, there are two general rules for constructing superhydrophobic materials and surfaces [34,35,36,37,38,39,40,41,42]: the coating should have an appropriate hierarchical surface structure with micro-nano dual scales, and the coating must have at least moderately low surface energy component, e.g., hydrocarbon or fluorocarbon compounds. There are various physical and chemical approaches to satisfy these two requirements to realize superhydrophobic abilities for cellulose-based substrates [43,44,45,46,47]. Textiles or membranes are generally believed to be rough substrates with reentrant curvatures and controllable diameter or spacing of fibers that are suitable for the achievement of super-antiwetting ability [48,49,50]. The most common techniques to construct rough coatings for the formation of superhydrophobic cellulose-based materials include various dip-coating methods, wet chemical etching, chemical bath deposition, electro-assistant deposition (electrophoretic deposition, electrospinning), spray-coating, chemical vapour deposition, and plasma processing techniques. Typically, these techniques can be divided into two categories, *viz*., wet chemical methods and dry physical techniques. Although most reports demonstrated that super-antiwetting surfaces with special wettability could been fabricated via a single method or process to successfully change sole surface structure/component or both of them simultaneously, dual/multiple processes are required to realize structural and chemical requirements in some specific cases [13,47].

The summary of most common synthesis techniques for superhydrophobic coatings on cellulose-based substrates is listed in Table 1. Detailed discussion on various processes to construct superhydrophobic coatings on cellulose-based substrates is presented in following sections under “Wet-Chemical Methods” and “Dry Methods”.

### 2.1. Wet Chemical Methods

#### 2.1.1. Dip-Coating Methods

Dip-coating to cover the fiber surface with a layer of hydrophobic inorganic micor/nano-particles, such as TiO_2_, SiO_2_ and ZnO, is the most common and versatile technique to construct a super-antiwetting coating on various textile substrates [51,52,53,54,55,56,57,58,59,60,61,62,63]. It typically requires at least three individual processing steps, e.g., dipping in the coating slurry, drying, and curing. Usually, the coating slurry contains organic solvent components that wet the textile and disperse particles, nano/micro dual scale particles components that increase the coating roughness, and specific polymer component that increase the binding strength. In some cases, the coating slurry may also contain hydrophobization agents, e.g., fluorocarbon silane, to decrease the surface energy of the coating layer. Some super-antiwetting fabrics constructed via the dip-coating process display surprisingly good mechanical durability attributing to the strong polymer binders and hierarchical roughness of stable particle coating.

**Figure 2:**
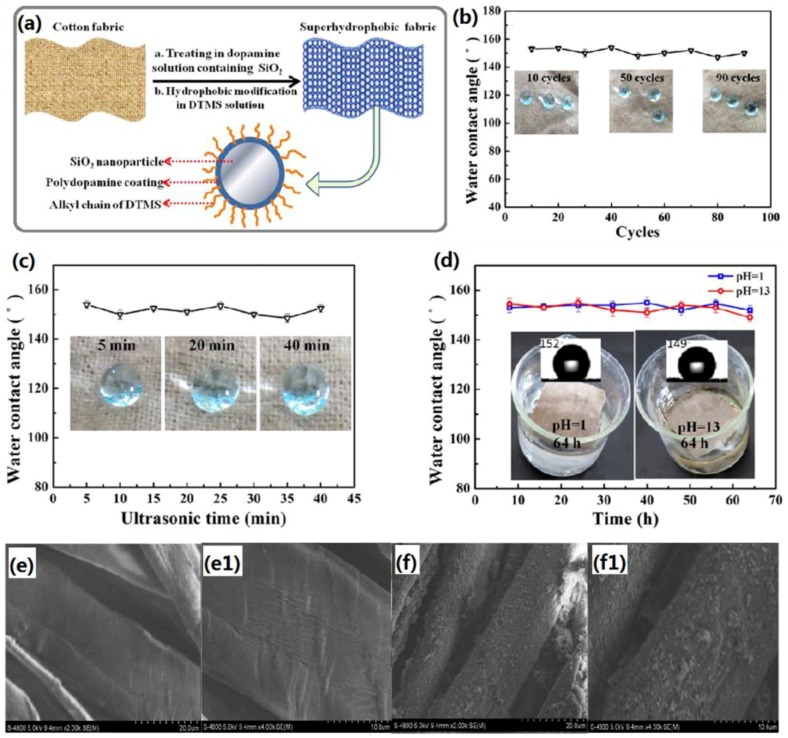
(**a**) Schematic representation of the preparation of superhydrophobic fabric; (**b**) Change of water contact angle of superhydrophobic fabric *versus* the recycling numbers of toluene/water mixture separation; (**c**) Change of water contact angles of superhydrophobic fabric after being ultrasonically treated for different time; (**d**) Change of water contact angles of superhydrophobic fabric after being exposed to acidic and alkaline solutions for different time; the morphology of (**e**,**e1**) pristine fabric and (**f**,**f1**) superhydrophobic fabric. (Reprinted from Reference [56] with permission).

Inspired by the amazing super-antiwetting ability of lotus leaf and the bioadhesion of mussel adhesive protein, Wang *et al.* fabricated superhydrophobic cotton fabric through the robust immobilization of SiO_2_ nanoparticles and subsequent hydrophobic modification [56]. The simple preparation process of superhydrophobic cotton fabric and the surface appearances of pristine and as-prepared fabric are shown in Figure 2. It is evident that the surface of pristine fabric is relatively smooth with intrinsic woven fabric structure, while the fabric surface is completely covered by a large number of nanoparticles after the treatment. Such rough microstructure can be full of air, thus preventing the penetration of water droplets into the cavities or interspaces of the fabric surface to improve the hydrophobicity. The obtained fabric shows high separation efficiency for a wide range of oil-water mixtures.

#### 2.1.2. Wet Chemical Etching

Chemical etching increases the surface roughness of the fibers substrates. In case of hydrophobized microscale cellulose membranes, a simple chemical etching can yield the surface super-antiwetting behaviour due to the wetting state transition from the Wenzel to Cassie-Baxter regime. It is well known that fluoropolymer is a kind of materials with low surface energy to form superhydrophobic surface, therefore a simple soaking process in reactant-containing solution would be a facile technique to achieve superhydrophobic property on substrates with appropriate surface roughness. For example, Wu *et al.* reported an extremely simple solution soaking coating in fluoropolymers (FPs) process for preparing extremely durable superhydrophobic textiles [64]. The textiles coated under the optimal conditions show excellent superhydrophobicity, mechanical (e.g., abrasion and laundering), environmental (e.g., UV irradiation, very low and high temperatures) and chemical (e.g., acid, base and organic solvents) stabilities.

**Figure 3:**
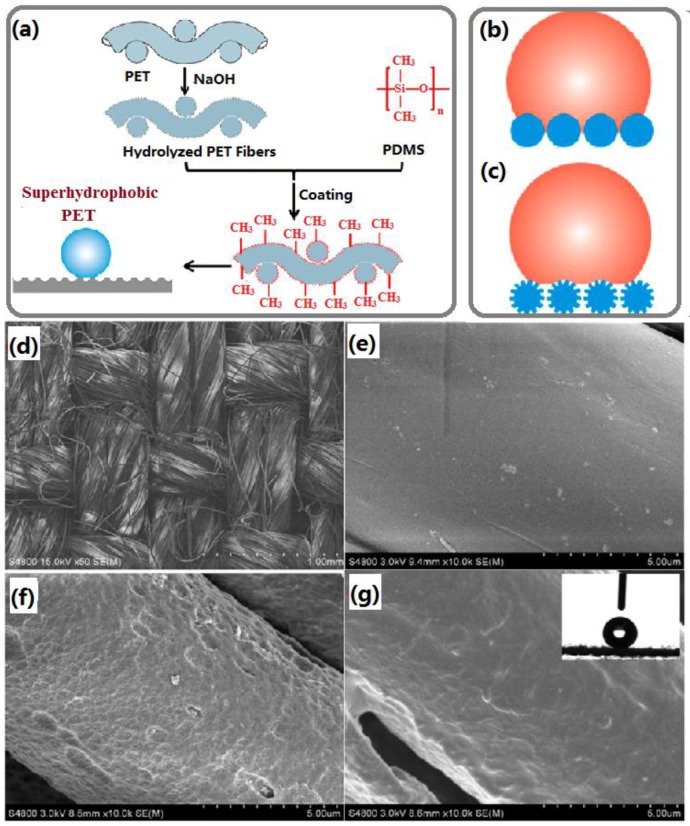
(**a**) Schematic illustration of the synthesis process of superhydrophobic textiles. Schematic models of a water droplet contact with hydrophobic textiles with smooth fibers (**b**) and superhydrophobic textiles with etched fibers (**c**); SEM images of (**d**) pristine poly(ethylene terephthalate) (PET) textile, (**e**) pristine PET fibers, (**f**) E-PET fibers, and (**g**) polydimethylsiloxane (PDMS)-E-PET with a PDMS add-on of 3 wt %. The inset of (**g**) is the optical image of a water droplet on the PDMS-E-PET surface. (Reprinted from Reference [68] with permission).

Xue *et al.* prepared colorful superhydrophobic poly(ethylene terephthalate) (PET) textile surfaces with self-cleaning property by chemical etching and coating with polydimethylsiloxane (PDMS) [68], as shown in Figure 3. Firstly, the original textiles were cleaned with deionized water to remove the impurities and dried. The cleaned textiles were dipped into sodium hydroxide solution for 10 min. Then the soaked textiles were doubled-side covered in a polyethylene film and heated at 120 °C. Finally, the textiles were rinsed by abundant water until the pH of the textile surfaces reached 7 and dried in an oven. Thus, chemically etched PET textiles were obtained and denoted as E-PET. After etching of the solid surfaces of PET fibers, pits were formed not only to decrease the contact area between the water droplets and textile surface, but also trap air for improving the fiber hydrophobicity, thus making water roll easily on the textiles. The as-obtained textiles possess remarkable durability against different pH solutions without changing its super-repellent feature and exhibit excellent resistance to washing, abrasion, even exposure to UV light. Importantly, colorful images could be imparted onto the superhydrophobic surfaces by conventional dyeing or thermal transfer printing on the textiles. The method is simple and requires no special equipment, which is suitable for large-scale production.

#### 2.1.3. Chemical Bath Deposition

**Figure 4:**
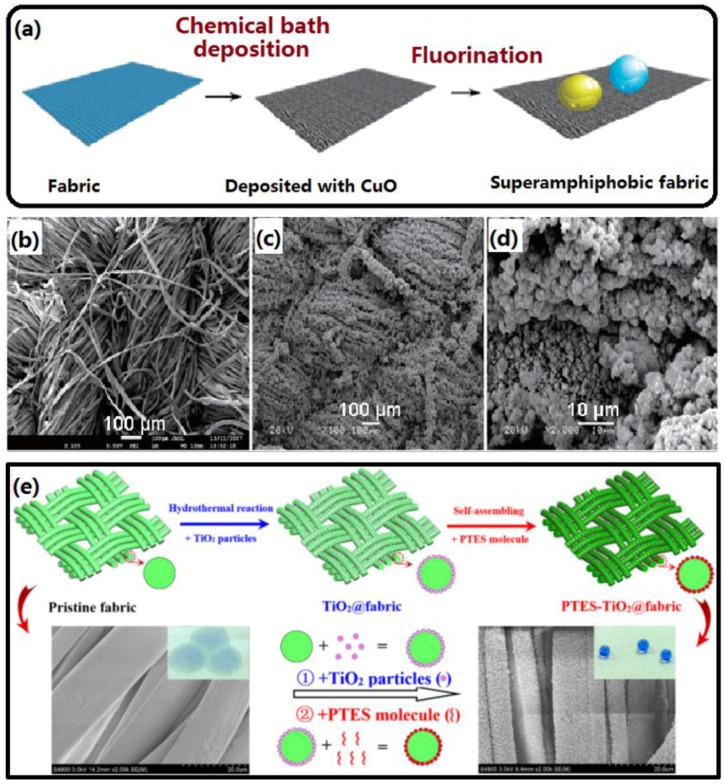
(**a**) Schematic diagram of the fabrication process for strong amphiphobic fabric; (**b**) SEM images of the original fabric; (**c,d**) Low- and high-magnified images of the CuO-coated fabrics (Reprinted from Reference [69] with permission); (**e**) Schematic illustration of the procedure used to construct superhydrophobic TiO_2_ particles-decorated cotton fabric and their corresponding SEM images and wetting behaviour before and after TiO_2_ particles-decoration and 1H, 1H, 2H, 2H-perfluorodecyltriethoxysilane modification. (Reprinted from Reference [70] with permission).

Zeng *et al.* reported a simple chemical bath deposition method to deposit CuO nanoparticles on fabric [69]. The as-prepared fabric exhibited superhydrophobicity and highly oleophobicity after a fluorination process as shown in Figure 4a–d. The as-obtained samples exhibited excellent self-cleaning properties for water and oils such as ethylene glycol and rapeseed oil. Huang *et al.* formed covalently bonded flower-like TiO_2_ nanoparticles on cotton fabrics by *in-situ* growth via a chemical bath deposition process [70], in which the cotton was immersed in the reaction mixture for several hours at 80 °C. Then a self-assembling process of fluoroalkylsilane was carried out to construct a robust superhydrophobic TiO_2_@fabric (Figure 4e). The obtained composite TiO_2_@fabric is promising to be adapted for the design of multifunctional fabrics with good anti-UV, effective self-cleaning, efficient oil-water separation, and microfluidic management applications. Zhang *et al.* have employed this method to prepare a robust ZnO film on a cotton surface to create rough structures [71]. After grafting with (heptadecfluoro-1,1,2,2-tetradecyl)trimethoxysilane with low surface energy, the wetting property of the fabric sample transformed from superhydrophilic to superhydrophobic with a WCA of 158°, and its LOI (limiting oxygen index) value greatly increased from 18.3% to 21.6%, showing its outstanding superhydrophobicity, flame retardancy and thermal stability. The above-mentioned superhydrophobic cotton fabrics constructed via wet chemical deposition exhibited outstanding antiwetting, UV shielding, durability and flame retardancy, offering an opportunity to accelerate the large-scale production of superhydrophobic textiles materials for new industrial applications.

#### 2.1.4. Electric-Field Assisted Etching/Deposition 

The electric-field assisted deposition technique, including electrophoretic deposition, electrospinning and electrostatic layer-by-layer assembly is a well-established industrial process that has been applied for fast and scalable deposition of large-scale films on conductive substrates in a stable suspension electrolyte [76,77,78,79]. Electrophoretic deposition (EPD) has been used to form homogeneous and stable TiO_2_-based nanobelt thin films [76]. Firstly, a stable titanate nanobelt particle suspension was prepared by a hydrogen-bond-driven assembly of pre-hydrolysed fluoroalkylsilane on its surface. Then a one-step electrophoretic deposition was applied to fabricate a transparent cross-aligned superhydrophobic TNB/FAS film on conducting substrates. Such a surface has displayed high chemical stability, self-cleaning ability and anti-fogging applications.

**Figure 5:**
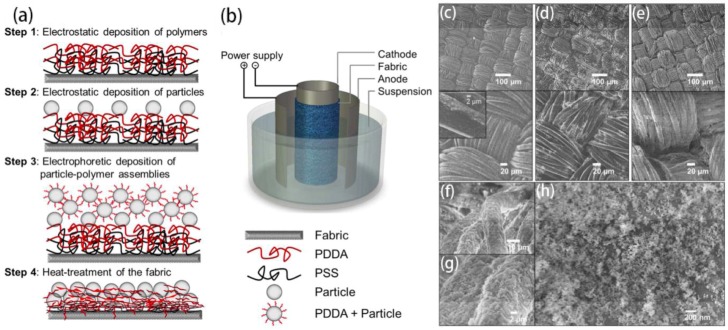
(**a**) Schematic illustrations of fabrication process suggested for anti-wetting fabric coating; (**b**) The electrophoretic deposition (EPD) cell for step 3 in (a). The cell consists for two stainless steel electrodes with the fabric substrate wrapped around the cathode electrode; (**c**) Original polyester fabric has twill weave patterns. The inset shows a fabric fiber with SiO_2_ nanoparticles deposited on the surface (after Step 2); (**d**) After 30 s of EPD, SiO_2_ nanoparticle layers are observed on the fabric but do not fully cover the surface; (**e**) After 60 s of EPD, the fabric fibers are uniformly and densely coated with SiO_2_ nanoparticles. (**f**–**h**) Magnified SEM images of the fabrics after 60 s of EPD (Reprinted from Reference [77] with permission).

Buie *et al.* coated polyester fabrics using electrostatic layer-by-layer (LBL) assembly of poly(diallyldimethyldiammonium chloride, poly(sodium 4-styrenesulfonate) and SiO_2_ nanoparticles firstly [77]. Then the fabrics were further coated with SiO_2_ particles-polymer assemblies by an EPD process. A superhydrophobic composite coating was realized after a heat treatment (Figure 5). This method had advantages of scalability, durability, and control of wettability, which also showed great potential for commercial use.

**Figure 6:**
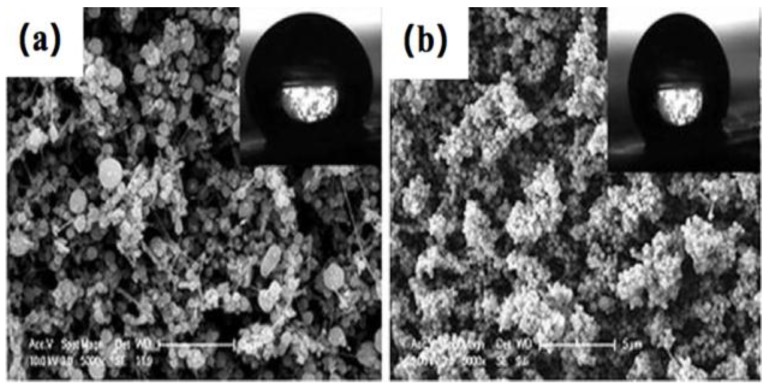

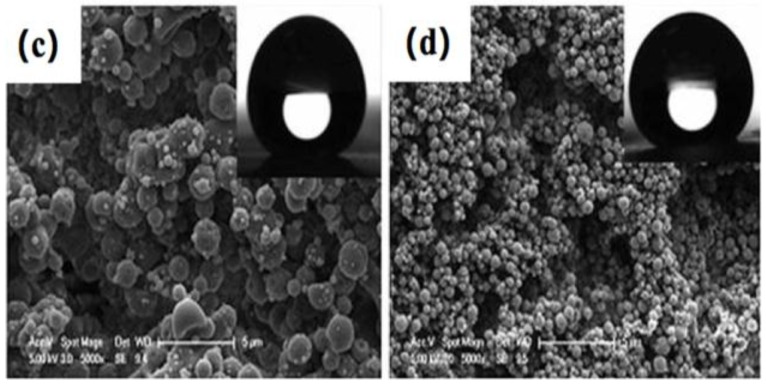
SEM images of various polyimide-siloxane mats through the electro-spinning of different poly(amic acid) precursor solutions synthesized by the condensation reaction of 3,3’,4,4’-benzophenone tetracarboxylic dianhydride (as a dianhydride) with a mixture of 4,4‘-oxydianiline and aminopropyl terminated polydimethylsiloxane (as diamines). (**a**): PIS-1, (**b**): PIS-2, (**c**): PIS-3 and (**d**): PIS-4 at 5000× magnification. (Reprinted from R eference [81] with permission).

Electro-spinning, another main electro-field assisted process, is a promising and straightforward technique that produces nano- and micro-fibers cross-stacked nonwoven materials [81,82,83,84,85,86,87]. Superhydrophobic polyimide-siloxane mats can be fabricated using an electrospinning process [81]. Firstly, to obtain the poly(amic acid) solutions with various siloxane content and siloxane block length, condensation reaction of diamine and dianhydride was performed. Afterward, PAA solutions were electrospun using an electrospinning process. Finally, thermal imidization was performed. Polyimide-siloxanes, which are abbreviated as PIS-1, PIS-2, PIS-3 and PIS-4 were successfully prepared and electrospun, as shown in Figure 6.

#### 2.1.5. Spray-Coating

Spray-coating is a facile, rapid and versatile way to build rough multi-scale hierarchal structure. It can be used to coat a layer of low surface energy polymer on all kinds of substrates [88,89,90,91,92,93,94,95]. For example, Yang *et al.* fabricated superhydrophobic/superoleophilic epoxy/attapulgite nanocomposite coatings on the stainless steel meshes by a simple spray-coating process. The authors demonstrated the coated mesh maintained highly superhydrophobic property after being treated in various harsh conditions, including mechanical scratch, high temperature, humid atmospheres, and corrosive substance [89].

#### 2.1.6. Other Wet Methods

**Figure 7:**
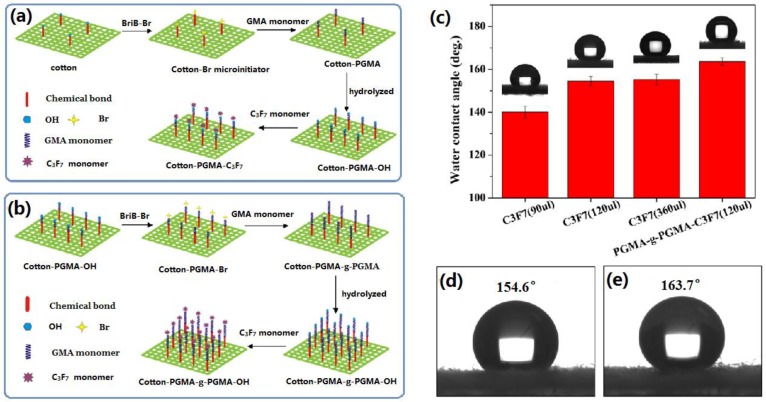
(**a**) Schematic diagram of synthetic approach for grafting fluoroalkyl chains on cotton surface; (**b**) Schematic diagram of synthetic functional cotton surface via graft-on-graft approach; (**c**) change of water contact angle on grafted functional cotton surface with different fluoroalkyl amount and graft-on-graft cotton surface. Insets are the images of water contact angle, respectively. The images of the water contact angle (CA) on C_3_F_7_ modified graft fabric surface (**d**) and graft-on-graft fabric surface (**e**). (Reprinted from Reference [111] with permission).

In most cases, long chain fluorine-based precursors were grafted for surface treatment to develop superhydrophobicity on the substrate surface. The safety and cost concerns of the long chain fluorine-based precursors greatly restrict such materials for scalable production. Recently, Lai *et al.* grafted short fluoroalkyl chain on cotton fabrics via a rational strategy to construct robust superhydrophobic fabrics with excellent air permeability behavior, good anti-wetting, and mechanical stability under multiple dry abrasion and wet laundering processes [111]. In this work, short fluoroalkyl chain of C_3_F_7_ with low energy compounds was robustly grafted on cotton fibers through an atom transfer radical polymerization (ATRP) strategy. A double graft-on-graft route has also been employed to enhance the capacity of maintaining its superhydrophobic, as shown in Figure 7a,b. Compared to the highest water CA on conventionally PGMA-grafted surfaces with saturated C_3_F_7_ chain termination (~155°), the graft-on-graft architecture surfaces exhibit a higher WCA of 163.7 ± 2.5° (Figure 7c,d). These results verify the designed graft-on-graft architecture is an effective and promising approach to achieve excellent super-anti-wetting ability with environmentally-friendly short fluoroalkyl chains. This chain grafting technique enables creation of superhydrophobic coatings in a sophisticated manner through closely controlled chemical reactions. Drawback of grafting techniques is their specific multi-step process and time-consuming nature. Zhang *et al.* also used wet chemical grafting polymerization to construct superhydrophobic polymeric films on the surface of cotton fabrics [112]. The superhydrophobic films were fabricated by immersing cotton fabrics in the siloxane solution followed by the treatment of low temperature plasma with glow discharge at a pressure of 10 Pa. Such simple, cost-effective, and environmental-friendly technique has a positive effect on the construction of fluorine-free superhydrophobic films on fabric surfaces [113].

Sol-gel processing is a well-recognized method of synthesizing gels and nanoparticles [114,115,116,117]. The surface roughness obtained with the sol-gel method can be easily tuned by changing the protocol of the method and the composition of the reaction mixture. Sol-gel process to fabricate superhydrophobic surface has been studied extensively over the past decades. Liu *et al.* performed a very simple one-step sol-gel approach to fabricate transparent and self-cleaning superhydrophobic coatings via the processing of long-chain fluoroalkylsiane [117]. The coating exhibited a rough, wrinkled, hill-like surface morphology, and the water drops assumed a spherical shape on this surface with a contact angle of 169° and a sliding angle of less than 5°. The prepared superhydrophobic coating exhibited an excellent self-cleaning performance. The superhydrophobic wetting state was well preserved even after the impact of a high-speed water jet. 

Zhu *et al.* reported a simple and rapid solvent swelling method to scale up the construction of superhydrophobic surface [118]. At first, the fabrics were swelled in cyclohexane/heptane mixture at 80 °C. Subsequently the recrystallization of the swollen macromolecules on the fiber surface contributes to the formation of submicron protuberances, which increase the surface roughness dramatically and result in superhydrophobic behavior. The as-obtained superhydrophobicity demonstrates excellent durability and robustness, such as good resistances to water penetration, abrasion, acidic/alkaline solution and boiling water.

### 2.2. Dry Methods

#### 2.2.1. Chemical Vapour Deposition

**Figure 8:**
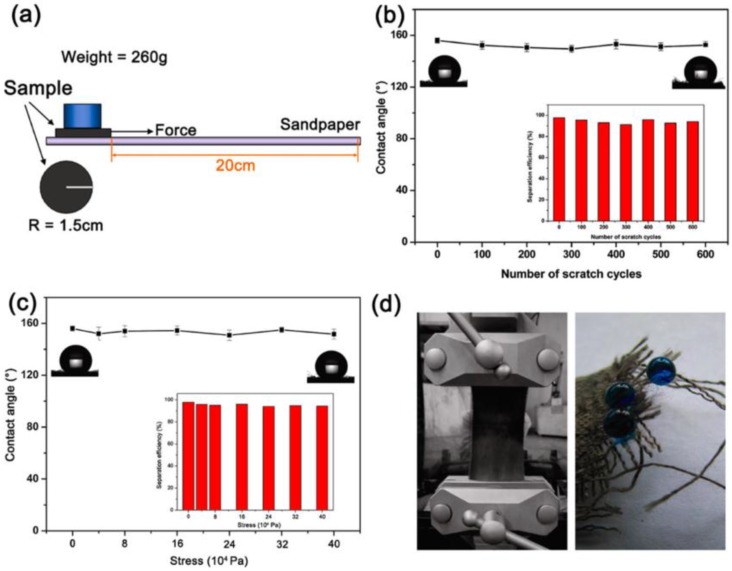
(**a**) Schematic illustration of the methodology of the abrasion test. The variation of water contact angles of the coated fabric (**b**) scratched by the sandpaper, and (**c**) stretched by axial tensile forces. Insets are the corresponding separation efficiencies for the oil-water mixture; (**d**) Images of the stretched textile (left) and water droplets on the broken yarns (right). (Reprinted from Reference [99] with permission).

Chemical vapor deposition (CVD) is a typical dry technique enable tuning of chemical and physical fine structure to be deposited onto a substrate in the form of nonvolatile film via the reaction of gaseous reactants [96,97,98,99,100,101]. Bao *et al.* developed a novel method for fabrication of superhydrophobic surfaces by facile coating various metal oxide nanoparticles, including ZnO, Al_2_O_3_ and Fe_3_O_4_, on the fabrics followed by treatment with polydimethylsiloxane (PDMS) via chemical vapor deposition (CVD) method [98]. The combination of the improved surface roughness generated from of the nanoparticles via CVD aggregation with the low surface-energy of silicon-coating originated from the thermal pyrolysis of PDMS would be responsible for the surface superhydrophobicity. This strategy may provide an inexpensive and new route to surperhydrophobic surfaces, which would be of technological significance for various practical applications especially for separation of oils or organic contaminates from water. Zhou *et al.* introduced the incorporation of polyaniline and fluorinated alkyl silane to the cotton fabric via a facile vapor phase deposition process [99]. The as-prepared fabric surface possessed both superhydrophobicity with the water contact angle of 156° and superoleophilicity with the oil contact angle of 0°. This fabric can be applied for oil/water separation. Moreover, compared with other materials for oil-water separation, the reported process was simple, time-saving, and repeatable for at least 30 times. Therefore, the as-prepared fabric exhibits a great durable property under extreme environmental conditions such as high temperature, high humidity, strong acidic or alkaline solutions, and mechanical forces (Figure 8).

#### 2.2.2. Plasma Etching Processing 

The dry plasma etching processing is important for the rough structure construction on some specific cellulose fiber (e.g., silk) because the mechanical properties of silk fibers submerged in water or solvent could decrease the binding strength of van der Waals force and hydrogen bonds among the chain segments [102]. Zheng *et al.* investigated the PVDF membranes bombarded with high-energy oxygen ions emitted by the microwave plasma to produce active groups on the surface [103]. After treating with oxygen plasma etching, PVDF membranes were placed in methyltrichlorosilane gas for 40–120 min grafting at 25 °C. The water contact angle and sliding angle on the created lotus-leaf-like PVDF membrane were 155° and 4°, exhibiting superhydrophobic property and self-cleaning property. The method of plasma etching processing was a cheap and easily implementable method to prepare the superhydrophobic surface [104,105,106,107,108,109,110].

**Figure 9:**
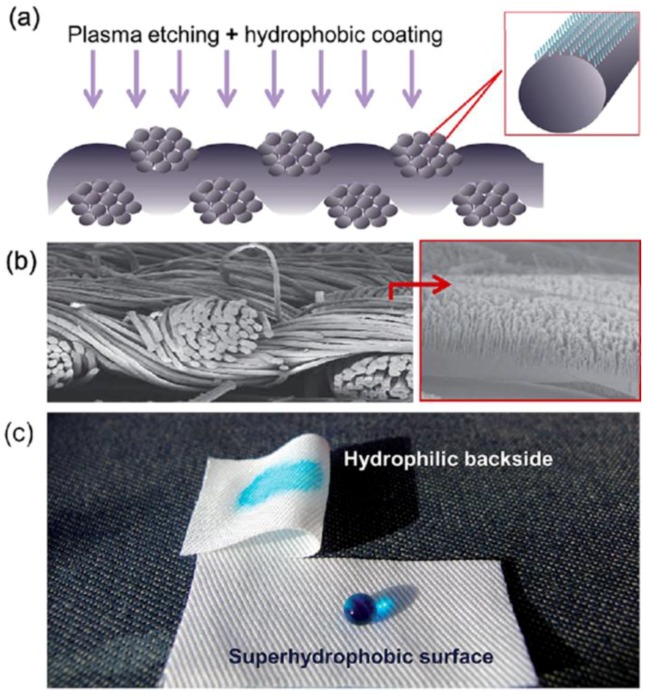
Nanostructured fabric surface: (**a**) schematic of plasma-processed fabric surface; (**b**) FE-SEM images of lyocell fabric that was oxygen etched for 20 min; (**c**) Photographic image demonstrating the asymmetric wetting behavior of the plasma-processed lyocell fabric. (Reprinted from Reference [107] with permission).

Kwon *et al.* employed oxygen plasma-based nanostructuring method with a subsequent coating with a low-surface-energy material to produce a single-faced superhydrophobic lyocell fabric maintaining its inherent high moisture absorbing bulk property (Figure 9) [107]. The achieved asymmetric wetting properties on a fabric layer would be significant and relevant for applications that require water repellency and self-cleaning properties, and simultaneously not compromising the clothing comfort.

#### 2.2.3. Other Dry Methods

**Figure 10:**
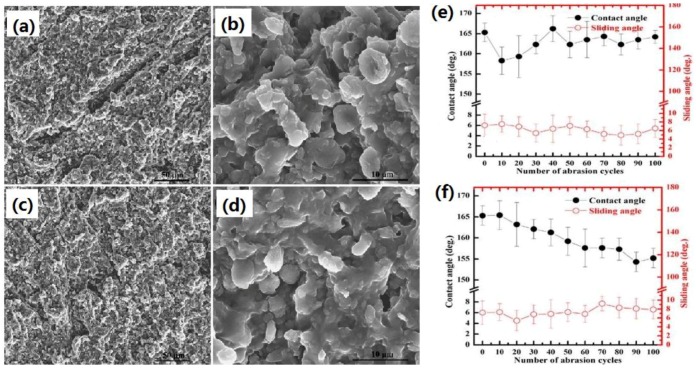
FE-SEM images of the surfaces of polytetrafluoroethylene/room temperature vulcanized silicone rubber (PTFE/RTVSR) composites after 100 abrasion cycles at different magnifications and WCA and WSA of the PTFE/RTVSR composite surface as a function of abrasion cycle. (**a**,**b**) 320# sand-paper was used as an abrasion surface; (**c**,**d**) cotton fabric was used as an abrasion surface; (**e**) 320# sand-paper was used as an abrasion surface; (**f**) cotton fabric was used as an abrasion surface. (Reprinted from Reference [122] with permission).

In addition to the above mentioned common techniques for the construction of superhydrophobic cellulose-based substrates, there are still some other specific methods that are suitable for the realization of super-antiwetting ability in certain fields, e.g., atomic layer deposition [119,120], plasma-assisted chemical vapour deposition [121], light irradiation grafting [122] or even structural change via abrading or physical transformation. For example, Xiao *et al.* successfully fabricated durable superhydrophobic wool fabrics with a coating of nanoscale Al_2_O_3_ layer by atomic layer deposition (ALD) [120]. The Al_2_O_3_ coating on wool fabrics changed the surface roughness and surface energy of fibers, resulting in increased static water contact angle from 130° to 160°. It was confirmed that ALD coating method could prepare superhydrophobic proteins to be used in applications the require water-proof and self-cleaning. Cortese *et al.* successfully prepared surfaces by one-step growth of a diamond-like carbon film onto textiles via plasma-enhanced chemical vapour deposition. Such film exhibited highly controllable, energy-efficient oil-water separation with high separation efficiency [121]. Wang *et al.* prepared polytetrafluoroethylene/room temperature vulcanized silicone rubber (PTFE/RTVSR) composites by a simple abrading method [122]. Cotton covered with these composites gained great mechanical durability because the elastic nature of the composite has enabled composites to avoid mechanical damage during the friction cycles (Figure 10).

## 3. Promising Applications of Superhydrophobic Fabrics

Recently, superhydrophobic fabrics have attracted great attention in various applications due to their non-stick and self-cleaning abilities. On the other hand, the smartly self-healing surface properties, and rational strategies for mechanical abrasion make these robust superhydrophobic fabrics more reliable for practical applications and commercialization in clothing industries. In this section, we mainly focus on the development of durable cellulose-based materials with superhydrophobic properties in oil-water separation, self-cleaning and asymmetric fabrics [123,124,125,126,127,128,129,130,131,132,133,134,135,136,137,138,139,140,141,142,143,144]. 

### 3.1. Oil-Water Separation

Nowadays, the increase of oily wastewater in industrial production and the frequent oil spill accidents has drawn worldwide attention. Besides the environmental problems, the contamination of oil, especially suspended oil to aquatic devices, has caused uncountable economic losses. Therefore, the removal and collection of the organic contaminants from water has attracted great attention. Here, we mainly focus on the introduction and discussion of the potential applications of porous membranes with superhydrophobicity for oil-water separation from simple oil-water layered mixtures to oil-water emulsions, and from non-intelligent membrane materials to intelligent membrane materials. Moreover, we will focus upon some of the latest rational strategies to prepare superhydrophobic cellulose-based membranes, which make them more effective, efficient and sustainable for oil-water separation.

**Figure 11:**
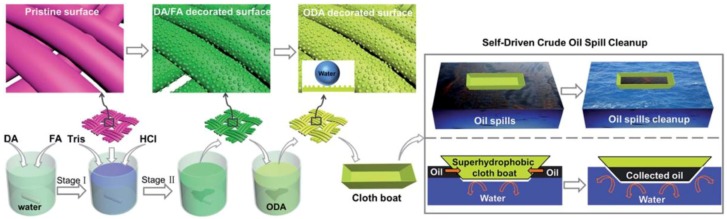
Schematic illustration of the fabrication of a superhydrophobic fabric with micro-nanostructures via a novel mussel-inspired approach and a superhydrophobic cloth boat made from the fabric for self-driven oil spill cleanup. (Reprinted from Reference [130] with permission).

Hydrophobic porous materials have gained tremendous interests because of their capability of selective absorption of oils/organic solvents while completely repelling water [129,130,131,132,133,134,135,136,137,138,139,140,141,142,143,144]. Inspired from mussel, Huang’ group fabricated the superhydrophobic sponge by decorating polydopamine nanoaggregates with n-dodecylthiol motifs on the skeletons of PU sponge [129]. These PU sponges are highly porous, super-antiwetting and mechanical durable. Shang *et al.* adopted electrospinning technology to prepare a nanofibrous membrane [130]. The membrane showed superhydrophobicity and superoleophilicity after modifying with fluorinated polybenzoxazine and silica nanoparticles, which endowed the membrane with good performance in water-oil separation (Figure 11). To improve the separation efficiency, a silicone modified hierarchically porous monolith was synthesized via a sol-gel and phase separation process, and was applied in cleaning oil away from water.

Taking into account the economic and environmental problems, Wang *et al.* realized a facile and versatile route for fabricating robust and rough polydopamine (PDA) coatings with hierarchical structures on the fabrics [131]. Their method does not need the incorporation of any additional nanoparticles. The hierarchical structure can be controlled by adjusting the concentrations of FA. After octadecylamine chemical manipulation, the as-prepared fabric exhibits excellent superhydrophobicity and superoleophilicity, as well as good stability. Self-driven and highly efficient crude oil spill cleanup can be achieved by the superhydrophobic fabric in the form of a boat, showing great potential for collection of oil spills and other organic chemicals from water surfaces.

Recently, Zhu *et al.* used three functional materials (copper mesh, fabric and sponge) for oil-water separation [132]. Samples were dipped into the solution of polyfluorowax-hydrophobic SiO_2_ to alter their surface texture and chemistry. This treatment endowed the samples with excellent superhydrophobic property, which could be used as a membrane to efficiently separate oil-water mixture. The as-obtained superhydrophobic spongesacted as an oil sorbent scaffold to selectively absorb oil from the oil-water mixture. More importantly, these superhydrophobic materials can retain high oil-water separation efficiency even after 10 cycles.

To achieve high oil-water separation effectiveness, Lai’s group prepared a TiO_2_@fabric composite for the marigold flower-like hierarchical TiO_2_ particles via a one-pot hydrothermal reaction on a cotton fabric surface [70]. After that, a robust superhydrophobic TiO_2_@fabric was realized by fluoroalkylsilane modification as an application for oil-water separation. Compared with hydrophobic cotton fabric, the TiO_2_@fabric exhibited an extremely high superhydrophobicity, which ensures highly efficient oil-water separation.

Based on absorption of SiO_2_ nanoparticles with subsequent heptadecafluoro-1,1,2,2-tetradecyl trimethoxysilane modification and heat treatment, Liang *et al.* transformed hydrophilic cellulose surfaces into extremely superhydrophobic ones in a facile way. In the process, SiO_2_ nanoparticles were covalently attached to the cellulose surface and fluorine containing siloxane coupling agent [134]. The creation of superhydrophobic, cellulose fabric-based materials and the potential applications in oil-water separation have also been investigated in the study.

Considering the oil cleanup, Song’s group suggested a benchtop prototype oil collection device by using selective wetting stainless steel mesh that simultaneously separates and collects the floating oil from water without the requirement of pre-separation pumping or pouring [135]. The collection efficiencies for oils with wide ranging kinematic viscosities are above 94%, including motor oil and heavy mineral oil showed high stability and functionality over repeated use.

Lei *et al.* developed a facile and inexpensive method for the fabrication of SiO_2_ nanoparticles functionalized with octadecyltrimethoxysilane which can be in-situ incorporated into cotton fabrics [137]. The prepared fabrics can be used to separate and capture a series of oils from water, like kerosene, toluene and chloroform, *etc.* The as-prepared fabrics showed robust and stable superhydrophobic properties towards hot water, many corrosive solutions (acidic, basic, salt liquids) and mechanical abrasion. Therefore, this reported fabric has the advantages of scalable fabrication, high separation efficiency, stable recyclability, and excellent durability, exhibiting great potential for industrial application.

As it is well-known that oil-water mixture will form a milky suspension after vigorous stirring, and will be separated into two layers after stewing. When solid particles are added, particles assemble on the oil-water interface, which prevents the oil phase from gathering together. This will lead to a relatively stable system of oil-water mixture. Recently, Dudchenko *et al.* demonstrated a method of membrane-based and fouling-free oil-water separation that couples carbon nanotube-poly underwater superoleophobic ultrafiltration membranes with magnetic pickering emulsions [138]. Its advantages are that it shows very high stability with the temperature change, and it is resistant to electrolyte, and stable in a wide range of pH solutions and polar solvents.

Above introduction of oil-water mixture belongs to simple oil-water layers, but when it comes to oil-water emulsion, the stable emulsion cannot be separated effectively by conventional methods such as floating, chemical coagulation, and thermal treatment. Addressing this challenge, membrane technology and phase inversion technique were proposed for the removal of stable oil/water emulsion (O/W). Arumugham and his co-workers designed a nano MgO/SPPSU/PPSU membrane that possesses good hydrophilicity by utilizing the sulfonated polyphenyl sulfone (SPPSU) as an anchoring agent as well as an interlayer modifying agent for oil removal from water (Figure 11) [140]. In castor O/W emulsion separation, the SPPSU and nano MgO were strongly immobilized by the electrostatic attraction. The anchored nano MgO hydrophilic particles showed great improvement in the membrane properties against O/W emulsion.

**Figure 12:**
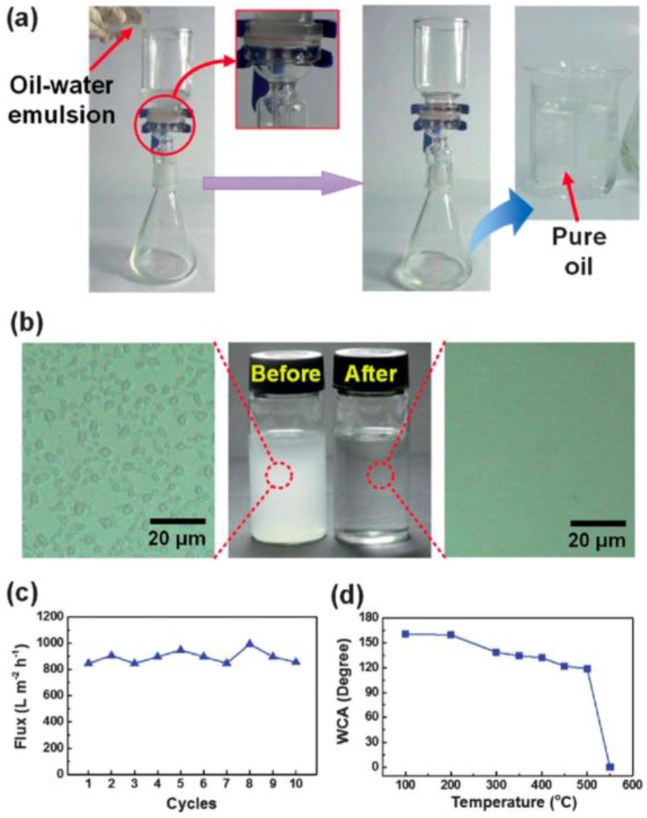
(**a**) The solely gravity-driven separation for oil-water emulsions using fluorinated silica nanofibrous (F-SNF)/Al_2_O_3_ membranes; (**b**) Photographs and optical micrographs of the oil-in-water emulsion before and after separation; (**c**) Change in the flux with increasing cycle number using F-SNF/Al_2_O_3_ membranes; (**d**) WCAs of the F-SNF/Al_2_O_3_ membrane after calcination at different temperatures for 5 min. (Reprinted from Reference [141] with permission).

Via *in-situ* polymerization, Huang *et al.* found a facile approach to fabricate superhydrophobic nonporous membranes prepared by polymerized 3-(3-triuoromethyl phenyl)-2*H*-benzoxazine-6-carbaldehyde. Effective separation of surfactant stabilized water-in-oil microemulsions was achieved [141]. Only driven by the gravity, this membrane showed high separation efficiency (with an extremely high flux of 892 L·m^-2^·h^-1^), as well as good antifouling properties, thermal stability and durability greater than those of commercial filtration membranes (Figure 12). More importantly, it matches well with the requirements for treating real emulsied wastewater on a mass scale.

**Figure 13:**
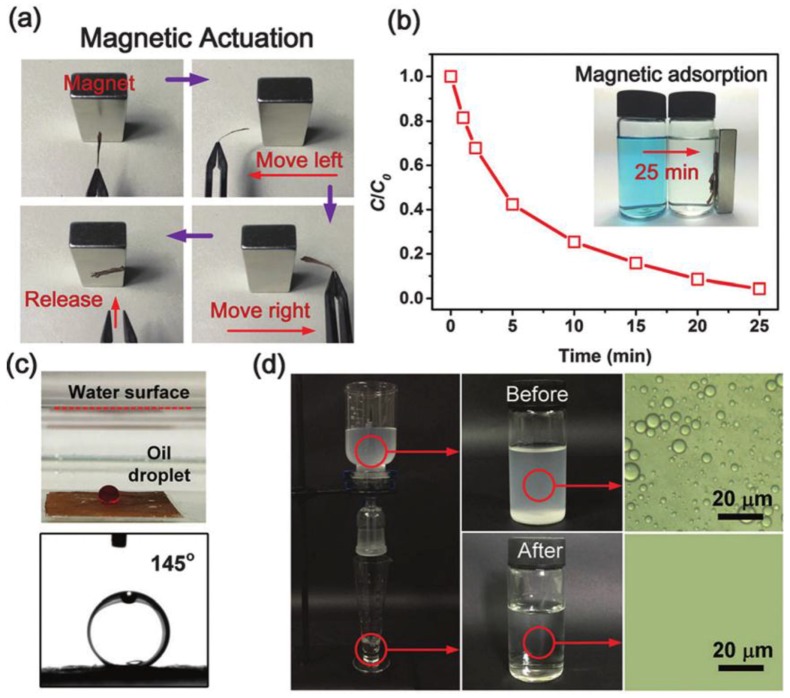
(**a**) A piece of NiFe_2_O_4_@Silica nanofibrous (SNF) membranes is held using tweezers upon a magnet, then the membrane bended towards the magnet when the tweezers move left or right, and it immediately flied to the magnet after being released; (**b**) The *C*/*C*_0_ *versus* time plots for the adsorption of dye solution, the inset shows the magnetic responsiveness of NiFe_2_O_4_@SNF after adsorption of the methylene blue (MB) for 25 min; (**c**) A photograph of an underwater oil droplet (dyed red) and the measurement of underwater oil contact angle on NiFe_2_O_4_@SNF; (**d**) The separation apparatus with the facile gravity-driven separation of oil-water emulsions using the NiFe_2_O_4_@SNF and the microscopic images of emulsions before and after separation. (Reprinted from Reference [143] with permission).

By combining electrospun nanofibers and the freeze-shaping technique, Ding’s group create fibrous, isotropically bonded and elastic reconstructed aerogels with a hierarchical cellular structure and superelasticity [142]. This approach intrinsically allows the lamellar deposited electrospun nanofibers to assemble into elastic bulk aerogels with tunable porous structure and wettability on a large scale. Using the gravity only, the fiber aerogels can effectively separate O/W emulsions with high flux and high separation efficiency. They also prepared flexible, hierarchical porous magnetic NiFe_2_O_4_@SiO_2_ nanofibrous (SNF) membranes by combining the gelatin method with electrospun nanofibers [143]. The NiFe_2_O_4_@SNF membranes exhibited prominent mechanical strength and mesoporosity, as well as multifunctionality of magnetic responsiveness, dye adsorption, and emulsion separation (Figure 13).

### 3.2. Self-Cleaning

Superhydrophobic and self-cleaning surfaces with a high static contact angle above 150° and low contact angle hysteresis play an important role in technical applications ranging from self-cleaning window glasses to paints and textiles, as well as low-friction surfaces for fluid flow and energy conservation. Self-cleaning materials are attracting more and more attention for its convenience and environment friendliness [145,146,147,148,149,150,151,152,153,154,155,156,157,158,159,160,161,162,163,164,165,166,167].

**Figure 14:**
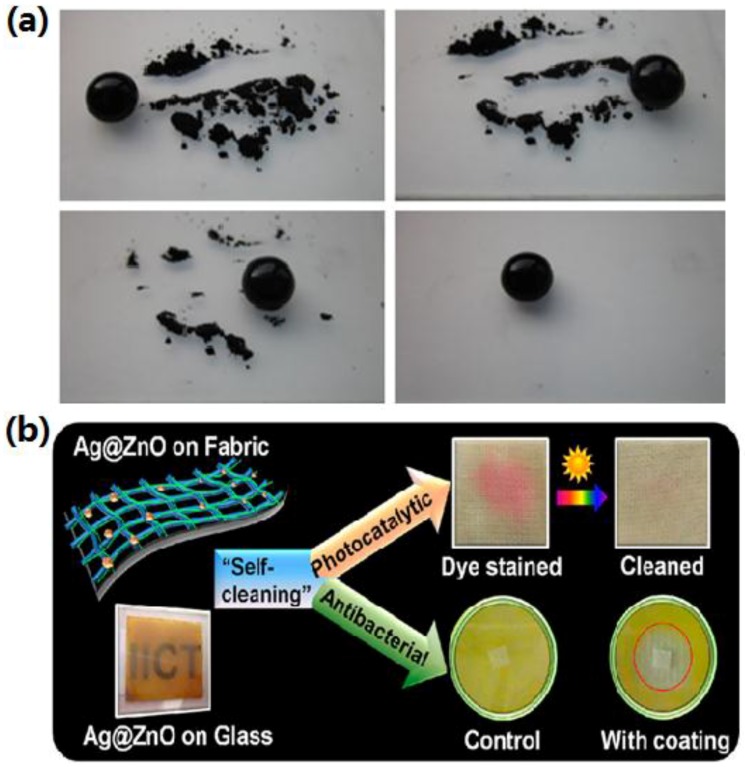
(**a**) Self-cleaning process on a superhydrophobic TiO_2_ surface (Reprinted from Reference [148] with permission); (**b**) Ag@ZnO nanostructured self-cleaning flexible materials with photocatalysis and anti-bacterial activities (Reprinted from Reference [150] with permission).

Mura *et al.* fabricated multifunctional textiles through the combination of the three different nanoparticles, the wool fabrics with the anchoring of TiO_2_, SiO_2_ and Ag nanoparticles exhibited good self-cleaning behavior for the removal of methylyene blue stain [147]. Pillai *et al.* tested the self-cleaning activities TiO_2_ film doped with metals or non-metals, the intrinsic photocatalytic activity of TiO_2_ film is beneficial for the self-cleaning activity. The topographical surface contaminated with organic matter or pollutants would be effectively cleaned by the TiO_2_ containing surface, attributing to the photocatalytic activity of TiO_2_ under UV light illumination [148]. Self-cleaning process on a superhydrophobic TiO_2_ surface was shown in Figure 14a.

Shahidi *et al.* used vacuum plasma apparatus treating the polyester fabrics with TiO_2_ anchored on the surface. The treatment has increased activation of the fabric surface and increased TiO_2_ absorption for more efficient and durable self-cleaning [149]. In addition to self-cleaning ability, Rana *et al.* reported that the Ag@ZnO nanostructures functionalized flexible cotton fabrics exhibited efficient visible-light photocatalysis and antibacterial activity [150] (Figure 14b).

Maintaining the long-term stability of superhydrophobic surfaces is challenging, especially for the UV-shielding fabrics loaded with semiconductor nanoparticles, e.g., TiO_2_ and ZnO, because the organic molecules and proteins contamination render the surface hydrophilic. Reactive oxygen species with high oxidation ability generated on a photocatalyst could mitigate to decompose these contaminants. However, incorporation of such catalyst particles into a superhydrophobic surface is challenging because the particles become hydrophilic under UV exposure, causing the surface to change to the Wenzel state.

**Figure 15:**
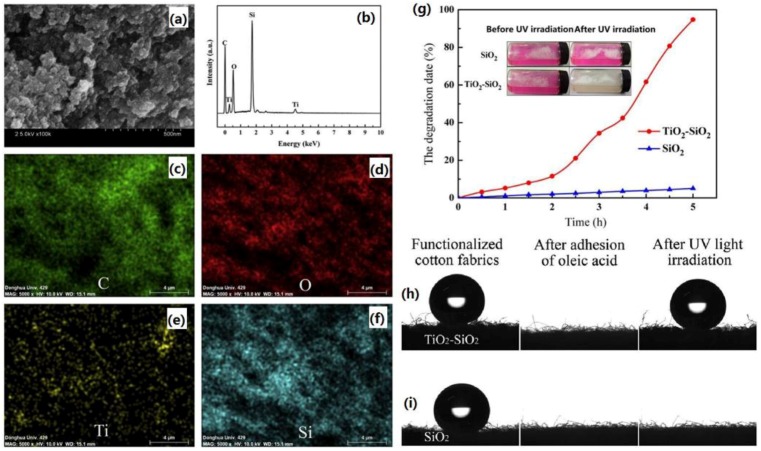
Typical SEM image (**a**); EDXS spectrum (**b**) and corresponding element mapping of the TiO_2_-SiO_2_ composite particles (**c**–**f**); (**g**) RhB solution photodegradation in the presence of ormosil aerogel or TiO_2_-SiO_2_ composite particles before and after UV irradiation (inserts: color changes of the solution before and after UV irradiation). Optical images of water droplets on (**h**) TiO_2_-SiO_2_ composite particle coated cotton fabrics and (**i**) ormosil aerogel particle coated cotton fabrics before and after adhesion of oleic acid and after UV irradiation for 3 h. (Reprinted from Reference [152] with permission).

Cai *et al.* combined photoactive TiO_2_ and superhydrophobic SiO_2_ by depositing TiO_2_ onto a nanostructured organically modified silica (ormosil) particle at low temperature, the resulting fluorine-free superhydrophobic cotton fabrics with TiO_2_-SiO_2_ composite particles decoration exhibit simultaneous superhydrophobicity and photocatalytic self-cleaning property (Figure 15) [152]. Organically modified silica (ormosil) aerogel with a high surface area and high porosity was first prepared. TiO_2_ nanocrystals were then synthesized and simultaneously deposited onto preformed porous ormosil aerogel at low temperature (below 100 °C) to obtain TiO_2_-SiO_2_ composite particles. The cotton fabrics coated with TiO_2_-SiO_2_ composite particles exhibit superhydrophobicity with a water contact angle of 160.5°. More importantly, the TiO_2_-SiO_2_ composite particle coated cotton fabric, which was contaminated with oleic acid, can recover its superhydrophobicity after UV irradiation for 4 h.

Tian *et al.* found flux scaly nanostructures provided the possibility of achieving a self-cleaning underwater superoleophobic surface due to the underwater superoleophobicity with ultralow adhesion [153]. Daoud *et al.* formed self-assembled monolayers of tetra(4-carboxyphenyl)porphyrin on TiO_2_-coated cotton by a simple post-adsorption method, followed by hydrophobization with trimethoxy(octadecyl)silane. The prepared cotton fabrics exhibited both superhydrophobic and visible-light photocatalytic activities, showing good potential for practical self-cleaning applications [155]. Khajavi and Berendjchi studied the effect of dicarboxylic acid chain length on the self-cleaning property of nano-TiO_2_-coated cotton samples. Samples treated with oxalic acid absorbed greater amounts of TiO_2_ nanoparticles and showed better self-cleaning properties [156].

Montazer *et al.* studied the photocatlytic removing rate of the methylene blue (MB) dye stained on fabrics, and found more titanium isopropoxide (TTIP) led to better self-cleaning activity possibly due to more TiO_2_ nanoparticles involved in photocatlytic reaction to clean the MB contaminated fabrics [157]. Karimi *et al.* loaded fabrics with graphene oxide by using a simple dip coating method, the graphene/titanium dioxide nanocomposites were obtained with chemical reduction using titanium trichloride, making the composited graphene/titanium dioxide modified fabrics an ideal self-cleaning candidate [158]. Nai *et al.* blended fluorinated polyhedral oligomeric silsesquioxanes (POSS) with poly(vinylidene fluoride (PVDF)/TiO_2_ by stirring overnight and the resultant solution was electrospun to obtain F-POSS/PVDF/TiO_2_ micron- and nanofibers with self-cleaning capacity [161]. Nazari *et al.* investigated cotton fabric treated with different amount of nano TiO_2_ and SrTiO_3_ at different pH values for self-cleaning purpose. The results demonstrated that cross-linking in acidic pH improves the grafting of nano materials and ratio of 0.2% (SrTiO_3_)/0.3% (TiO_2_) has the best effect on discoloration performance [163]. Xue *et al.* demonstrated that washable and wear-resistant superhydrophobic colorful surfaces with self-cleaning property can be successfully constructed on PET textiles by chemical etching of the fiber surfaces followed by coating with PDMS [164].

**Figure 16:**
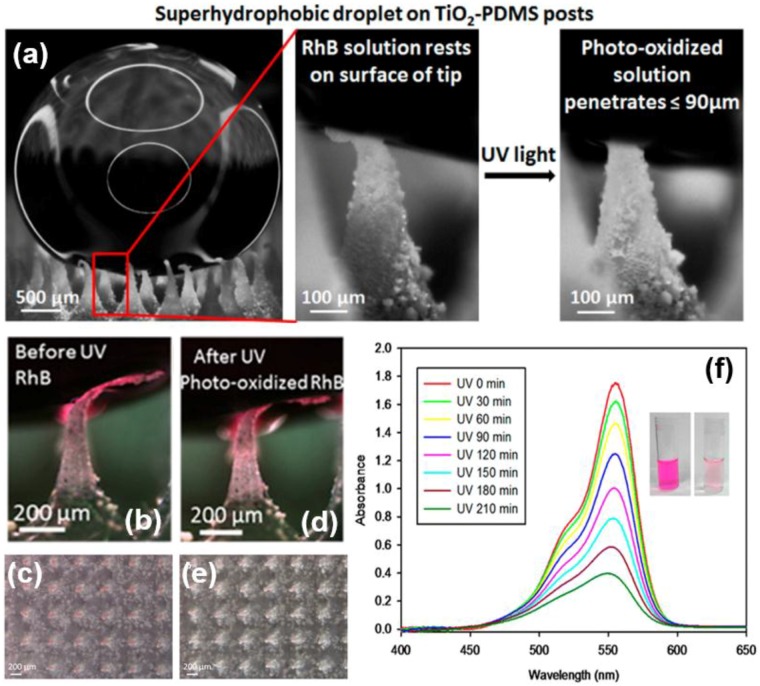
(**a**) A droplet on superhydrophobic TiO_2_-PDMS post and its enlarged wetting situation of a single PDMS post at liquid/solid interface before or after UV light photo-oxidation water process for 3 h. Close-up images of a single post and post arrays which partially supports a 15 μL droplet of 10 mg/L RhB solution before (**b**,**c**) and after (**d**,**e**) UV irradiation for 2 h; (**f**) UV-vis spectrum of the RhB solution supported on a TiO_2_-PDMS surface in the photoreactor after irradiation times. Inset pictures are the rhodamine B solution before (left) and after (right) UV photodegradation. (Reprinted from Reference [166] with permission).

Barletta *et al.* designed plastic fabrics based on a silicone-modified 1-pack polyurethane resin cross-linked with diisocyanates and promoted with nano-sized TiO_2_ powders and Ag ions supported on nano-sized ultra-porous glass-spheres. The fabrics have the most promising prospect for self-cleaning performance [165]. Lyons *et al.* described a self-cleaning superhydrophobic PDMS post arrays partially embedded with TiO_2_ nanoparticles. The composited surface maintained superhydrophobicity with a typical Cassie wetting state even when the hydrophilic TiO_2_ nanoparticles were embedded, and exhibited good super-antiwetting property after a long-term UV light irradiation (Figure 16) [166]. However, the conjugated dye, rhodamine B, and a bovine serum albumin protein were efficiently color removed or photo-oxidized by the inherent UV photocatalytic degradation ability of anatase TiO_2_ nanostructure materials [167].

### 3.3. Asymmetric Superhydrophobic Fabrics

Fabrics with asymmetric wetting behavior exhibit unidirectional water wetting behaviour, *i.e.*, liquid droplets are repelled from one surface with super-antiwetting property, while liquid droplets are absorbed on the other side. Achieving the asymmetric wetting properties is significant for function engineering to construct breathable, comfortable, self-cleaning, and less skin-irritating fabrics [168,169,170,171,172,173,174,175,176,177,178,179]. Wang *et al.* adopted a graft-polymerization process with atomized lauryl methacrylate as monomer to fabricate fluorine-less and asymmetrically superhydrophobic cotton fabrics [168]. The modified cotton fabrics exhibit laundering-durability and mechanically stability. The damages on the cotton fibers caused by solvent can be reduced at a very low level in the present process. Xi *et al.* employed a mist copolymerization of three monomers to fabricate asymmetrically superhydrophobic cotton fabric [169]. The modified cotton fabrics exhibit superhydrophobicity on one side but retain the inherent hydrophilic nature of cotton on the opposite side. Meanwhile, the modified cotton products show good water-absorbing ability and vapor transmissibility.

Liu *et al.* reported a new type of functional cotton fabric with single-faced superhydrophobicity achieved through a simple foam finishing process [170]. Fabric materials with such asymmetric and tailored wettability will be of great significance in textile, medical, and industrial applications, including microfluidic systems, desalination of seawater, flow management in fuel cells, and water-oil separation.

**Figure 17:**
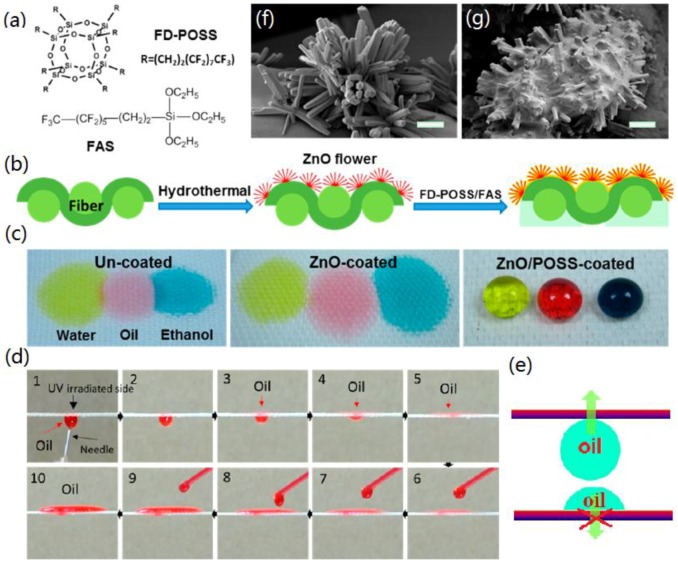
(**a**) Chemical structures of the coating materials; (**b**) coating procedure; (**c**) photographs of yellow-colored water, red-colored cooking oil, and blue-colored ethanol (volume 35 μL) on cotton fabrics; Still frames taken from a video to show the dropping of red-dyed cooking oil onto the UV-irradiated “front” and “back” surfaces (**d**) and corresponding schematic illustations (**e**); (**f**) after ZnO treatment and (**g**) after both ZnO and fluorinated-decyl polyhedral oligomeric silsesquioxanes (FD-POSS)/fluorinated alkylsilane (FAS) treatment (scale bar in the SEM images, 2 μm). (Reprinted from Reference [174] with permission).

Wang *et al.* reported the fabrication of asymmetric wetting polyester fabrics by a versatile method based on the combination of dip-coating process to create sueperhydrophobicity and UV exposure to obtain irradiated side hydrophilic, leading to the construction of unidirectional wetting fabrics [172]. Such asymmetric wetting fabrics had the ability to spontaneously transfer water unidirectionally through the fibrous architecture. The directional water-transfer fabrics should be able to remove sweat effectively from the body side, which is very useful for sportswear, soldier’s clothing, and daily life applications. Liu *et al.* used a two-layer self-assembly method [173]. A smart stimuli-responsive superhydrophobic surface based on the hierarchical structure of graphene and TiO_2_ nanofilm with bioinspired dual roughness, was constructed and applied onto the cotton fabrics. The surface exhibits tunable wetting, adhesion, and directional water transport properties, which provides a general protocol for applications such as moisture management, microfluidic control, self-cleaning, and water-oil separation. Wang *et al.* reported a novel method to prepare one-way oil-transport fabrics and their application in detecting liquid surface tension (Figure 17) [174]. This functional fabric was prepared by a two-step coating process to apply flowerlike ZnO nanorods, fluorinated decyl polyhedral oligomeric silsesquioxanes, and hydrolyzed fluorinated alkylsilane on a fabric substrate.

**Figure 18:**
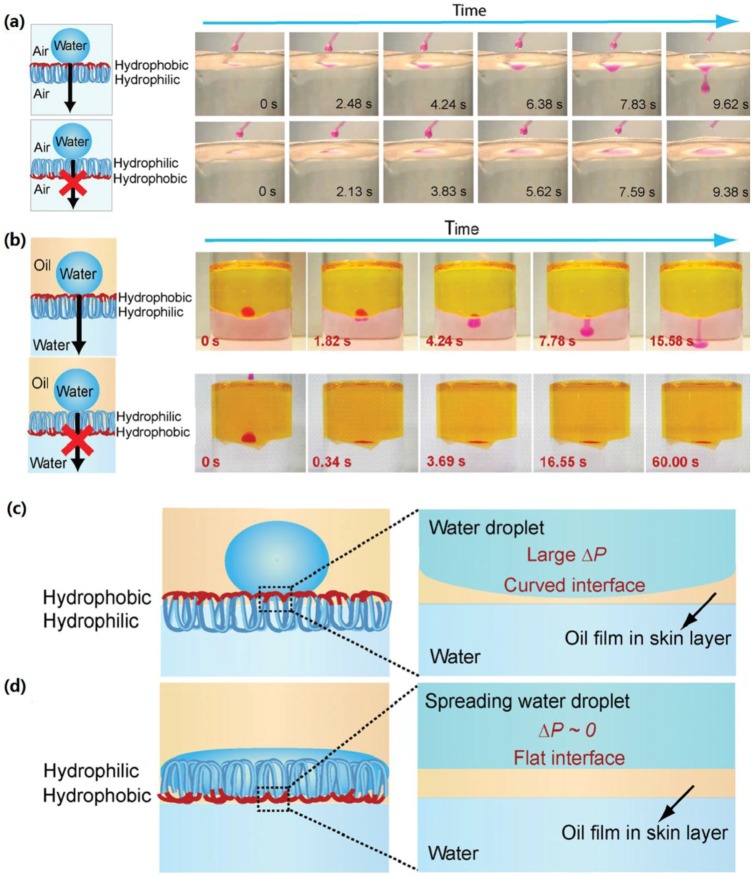
(**a**) Unidirectional droplet penetration demonstrated by dropwise addition of water droplets (droplet dyed red with rhodamine 101) onto hydrophobic side (top) and hydrophilic side (bottom) of Janus-C in air-water systems; (**b**) Janus-C allows penetration of water droplet (dyed red) when the hydrophobic side is towards oil (hexadecane dyed yellow by Nile red), and prevents droplet penetration when reversely aligned in oil-water systems; (**c**) For positively aligned Janus-C, a water droplet touching the hydrophobic side exerts a larger Laplace pressure (Δ*P*), creating a larger driving force for penetration. Consequently, the water droplet can penetrate through the thin oil-infused skin layer and further across the whole membrane; (**d**) For reversely aligned Janus-C, a water droplet touching the hydrophilic side tends to spread, exerting limited Laplace pressure. The oil-infused skin layer is thus able to block its penetration. (Reprinted from Reference [179] with permission).

Tian *et al.* prepared hydrophilic/hydrophobic Janus-type membranes involving chemically asymmetric skin-layer structures by facile vapor diffusion or plasma treatments. They have also discussed the corresponding droplet gating mechanism under various conditions [179]. As shown in Figure 18, the resultant Janus membrane shows directional water droplet gating behavior in air-water systems. Additionally, in oil-water systems, the Janus membranes show directional gating of droplets with integrated selectivity for either oil or water. The above remarkable gating properties of the Janus membranes could bring about novel applications in fluid rectifying, microchemical reaction manipulation, advanced separation, biomedical materials and smart textiles.

### 3.4. UV-Shielding/Durable and Self-Healing Superhydrophobic Fabrics

Recently, highly robust, durable, breathable, self-healing and switchable surface properties have attracted considerable attention to the production of superhydrophobic surfaces [180,181,182,183,184,185,186,187,188,189,190]. The above properties are very important for the commercialisation of materials for practical applications. On the other hand, the development of these properties is quite difficult. Therefore, rational strategies are needed when designing and fabricating these multifunctional surfaces with excellent properties. Superhydrophobic surfaces are responsive and switchable under circumstances, such as pH, light (UV, plasma, and laser), temperature, humidity and electrochemical treatments. All of these depend on the type of materials utilized for coating applications [191,192,193,194,195,196,197]. Moreover, the durability and breathability of anti-wetting surface is an important question we must address in order to broaden its practical applications. There are several areas to improve its durability including enhancement of mechanical stability, improvement of corrosion-resistance, and incorporation of responsive self-healing. Especially the self-healing ability responding to external stimuli is highly desirable for sustainable application [198].

UV-shielding durability is a fundamental challenge with superhydrophobic surfaces. However, recently there has been major progress in fabrication of robust superhydrophobic surfaces. For example, Xue *et al.* reported UV-durable superhydrophobic and UV-shielding PET fibers with ZnO/SiO_2_ core/shell structures by successive coating of multilayer polyelectrolytes [180,181]. The coating of silica not only improved the UV-shielding property but also extended the UV-durability of the superhydrophobic textiles.

**Figure 19:**
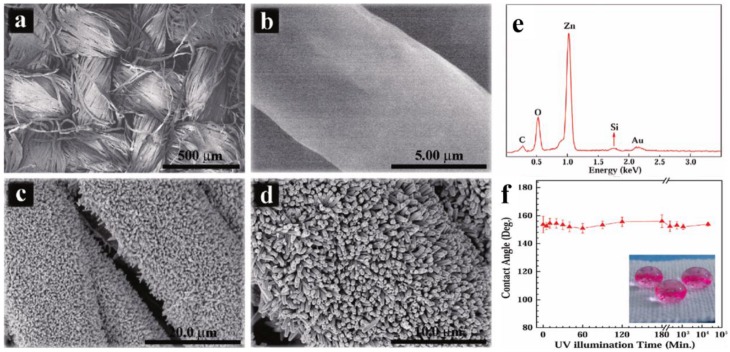
SEM images of native (**a**,**b**) and ZnO nanorod array coated (**c**,**d**) cotton textiles; (**e**) EDX spectrum of the sample corresponding to image (**e**); (**f**) Evolution of water CA on an OTS-modified ZnO@SiO_2_ nanorodarray coated cotton textile under UV irradiation (25 mW·cm^−1^). The *x*-axis after the break is scaled logarithmically. The inset is a macroscopic view of water droplets on the surface of a UV-irradiated sample. (Reprinted from Reference [185] with permission).

Wang *et al.* coated cotton textile with ZnO@SiO_2_ nanorods in mild conditions (Figure 19) [185]. Uniform ZnO nanorods were firstly grown on textile through a hydrothermal process, and then a SiO_2_ shell was coated on the surface of a ZnO nanorod by a bioinspired layer-by-layer deposition method. Finally, the ZnO@SiO_2_ nanorods coated cotton textile were modified with octadecyltrimethoxysilane to achieve superhydrophobic property. The as-prepared cotton textile exhibited an excellent UV-durable super-antiwetting property due to the protection of a UV shielding SiO_2_ layer.

**Figure 20:**
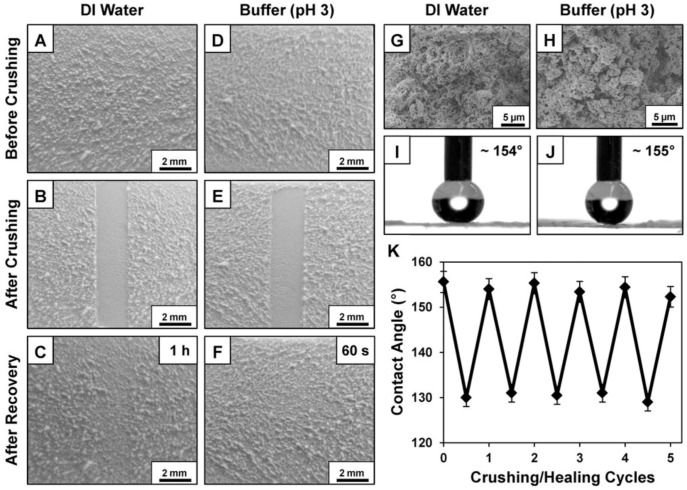
(**A**–**F**) Topographic features and wetting situation on self-healing cellulose-based surface. Top-down views of films before (**A,D**) and after (**B,E**) crushing, and (**C,F**) after immersion in deionized water for 1 h (**C**) or low-pH buffer for 60 s (**D**); films were dried under vacuum before imaging. SEM images (**G**–**H**) and contact angles (**I**–**J**) of the water-treated films shown in (**C**,**F**) in regions of film that were crushed/recovered, respectively. (**K**) Plot of contact angle *vs.* number of crushing/healing cycles applied. (Reprinted from Reference [203] with permission).

Recently, self-healing functions have also been successfully incorporated into smart superhydrophobic surfaces [199,200,201,202,203]. One of self-healing route is the recovery of the topographic structures. This method is bioinspired by the living organisms and other living things [199]. Li *et al.* used poly(allylamine hydrochloride), sulfonated poly(ether ether ketone) and poly(acrylicacid) as precursors to fabricate micro/nano-scaled hierarchical structures through dipping layer-by-layer assembly [200]. After chemical vapor deposition (CVD) of a fluoroalkylsilane, these coatings become superhydrophobic. The self-healing can occur at slightly humid environment when the coating is decomposed or scratched. Recently, Li *et al.* further improved their synthesis method by using spraying LBL assembly to take the place of dipping LBL assembly during the preparation process [201]. In this process, they can also avoid CVD process by using perfluorooctanesulfonic acid lithium salt and 1H,1H,2H,2H-perfluorooctyltriethoxysilane (POTS) as healing agents. All of these make it more applicable. Moreover, the original super-antiwetting ability can be reversibly realized by simply re-spraying POTS solution. Recently, Manna *et al.* reported an approach to the self-healing in crushed polymer-based superhydrophobic coatings [203]. They applied crushing loads on porous superhydrophobic films to compact the coatings and flatten the micro/nanoscale features. The surface exhibited self-healing performance when treated by liquid water (Figure 20).

The second self-healing strategy is to repair surface chemical components [204,205,206,207,208,209,210]. Store some components as agent and they will migrate to the surface with special treatment. For example, Shillingford *et al.* presented a new method by using lubricant to replace air in the traditional superhydrophobic surfaces [204]. The cotton and polyester fabric treated by this slippery lubricant-infused porous surfaces function show splendid omni-repellent properties against various fluids including polar and nonpolar liquids, pressure tolerance and mechanical robustness properties. Chen *et al.* fabricated a cotton fabric with flame-retardant and self-healing superhydrophobic coatings by a convenient solution-dipping method [205]. After being exposed to flame, this tri-layer generated a porous char layer, and exhibited an outstanding self-healing property because the hydrophilic ammonium polyphosphate and branched poly(ethylenimine) coating expel the embedded hydrophobic perfluorodecanyl chains of the fluorinated-decyl polyhedral oligomeric silsesquioxane (FD-POSS) to speed up its migration to the coating surface.

Xue *et al.* sprayed polystyrene/SiO_2_ core/shell nanoparticles as a coating skeleton and polydimethylsiloxane as hydrophobic interconnection to create superhydrophobic surfaces [206]. After bring damaged from an air plasma treatment, the superhydrophobicity of the surface can recover in 12 h at room temperature or by heat curing and tetrahydrofuran treatment. Si *et al.* designed a green supehydrophobic gel nano-coating [207], which is easy to apply to all kinds of substrates through a spray method. They used cotton as substrate to test its self-healing property, and found that it can self-heal rapidly by using usual organic solvents such as acetone. Esteves *et al.* prepared a self-repairing coating from an all-in-one dispersion by a simple drop-cast method [209]. The self-healing can occur at room temperature and the sueperhydrophobicity would remain after 500 abrasion cycles.

Wu *et al.* reported a new method to prepare superhydrophobic cotton fabric with self-healing property using radiation-induced graft polymerization of lauryl methacrylate and n-hexyl methacrylate [210]. The self-healing property can be achieved by ironing, and the self-cleaning fabrics made from lauryl methacrylate grafted cotton fabric can ultimately withstand at least 24,000 cycles of abrasion with periodic steam ironing. This is because of the migration of the polymethacrylates graft chains from the interior to the surface after the ironing. Wang *et al.* reported that fabrics coated with a hydrolysis product from FD-POSS and a fluorinated alkylsilane (FAS) have a self-healing superhydrophobic surface [211]. It also showed excellent durability when exposed to acid, UV light, machine wash, and abrasion. The self-healing mechanism of the FD-POSS/FAS coating was explained to be molecular rotation and movement which could change the surface free energy when heated.

### 3.5. Other Potential Applications with Special Properties

A superhydrophobic coating on cellulose-based paper brings new properties and functionalities for the materials and widens their utilization potential in new application areas. Applications for multifunctioanl superhydrophobic fabrics and paper include liquid/dirt-repellent, breathable, self-cleaning, UV-shielding, super-buoyancy, corrosion-resistant, and anti-biofouling/bacterial clothes and textiles. In addition, superhydrophobic paper and fabrics have good utilization potential in some other important applications such as disposable/flexible microfluidic devices and packaging materials for, for example, point-of-care diagnostics or colorimetric detection of specific gas and bovine serum albumin [212,213,214,215].

**Figure 21:**
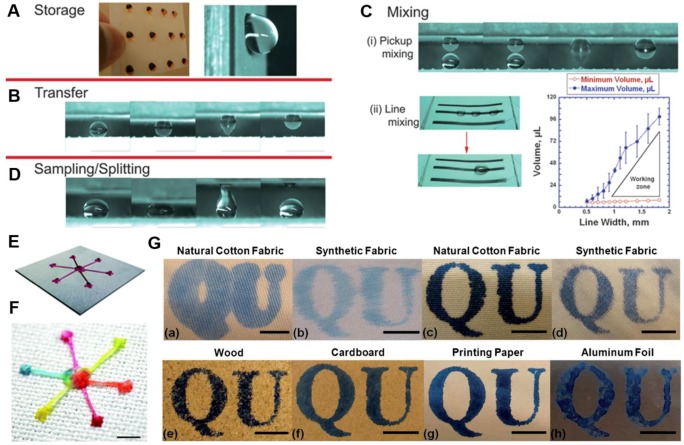
(**A**) Photographs of an array of drops (food coloring was added to enhance contrast) and a high magnification image of a single drop stored on a vertical substrate; (**B**) a series of snapshots of a drop being transferred between two substrates; (**C**) photographs of merging and mixing: (i) via “pickup mixing” (two drops), (ii) “line mixing” (three drops) and plot that shows the working zone of drop volumes suitable for line mixing; (**D**) photographs of drop splitting between two substrates. (Reprinted from Reference [220] with permission); A multi-inlet–single-outlet schematic illustration (**E**) and corresponding optical image (**F**) on the micropatterned superhydrophobic textile platform using the autonomous interfacial transport concept (note: aqueous solution at each inlet is colour-dyed for clear observation; scale bar: 5 mm). (Reprinted from Reference [222] with permission); (**G**) Patterns of “QU” that had been printed using the diluted ink onto various substrate (a) cotton fabric and (b) semi-synthetic cotton fabric (65% polyester/35% cotton). The rest of the photographs were taken of the pattern printed using the poly(ethylene oxide)-containing ink onto (c) cotton, (d) semi-synthetic cotton, (e) wood, (f) cardboard, (g) printing paper and (h) aluminum foil. The scale bars represent 1.0 cm. (Reprinted from Reference [224] with permission).

Surface wettability/adhesion micro-patterning on superhydrophobic surfaces created by, for example, UV light or laser [216,217,218] high energy ink/yarn [219,220,221,222,223] or low energy wax [212] offers possibilities in guiding the manipulation and transportation of liquid droplets and micro-flows, or in introducing the site-selective deposition of functional materials on the hydrophilic regions of the surface [224,225,226]. Breedveld *et al.* [220] fabricated two-dimensional paper-based lab-on-a-chip microfluidic devices by printing high surface energy black ink patterns on superhydrophobic paper substrate. The ink patterns provide the tunability of the wetting adhesion on the superhydrophobic paper, and thus enable the implementation of four basic operations for the manipulation of liquid drops on the paper substrates ranging from storage, transfer, mixing to sampling (Figure 21A–D). In their lab-on-paper prototype, liquid droplets or micro-fluids adhere to the porous substrate, rather than absorbing into the pater; as a result, they remain accessible for further quantitative detecting and analysis after performing simple qualitative on-chip testing. Xing *et al.* introduced a novel interfacial microfluidic transport principle to design a multi-inlet-single-outlet device on a micropatterned superhydrophobic textile (MST) for driving three-dimensional liquid flows in a more autonomous and controllable style (Figure 21E,F) [222]. In-proof-of-concept, the designed platform on MST has been applied on an artificial skin surface to collect and remove sweat in a highly efficient and facilitated means. The results have demonstrated that the novel interfacial transport strategy based on the textile platform are promising for biofluidic manipulations. Wang *et al.* yielded hydrophilically patterned superhydrophobic cotton fabrics via a site-selective photolysis reaction to anchor poly[2-(cinnamoyloxy)ethyl acrylate] block of the copolymer in the exposed region to crosslink around the cotton fibers [224]. While water-based solutions such as ink readily permeated the hydrophilic regions, they were blocked in the superhydrophobic regions. Thus, ink or dye reservoirs held by these wetting patterned cotton swatches were used as stamps for printing on various substrates, e.g., fabrics, cardboard, paper, wood, and aluminum foil (Figure 21G).

## 4. Summary and Future Perspectives

The present review deals with recent advances in research and promising applications for robust superhydrophobic cellulose-based materials. The various surface treatment techniques and design strategies are inspired by many biological systems. The main principle is to construct suitable rough structures with low surface energy to render super-antiwetting properties. The techniques used include dip-coating, chemical bath deposition, electric-field assisted coating, spray-coating, sol-gel *etc.* Some emerging promising applications for these flexible and robust super-antiwetting fabrics including oil-water separation, self-cleaning and anisotropic wetting are reviewed in this article. The wide ranges of applications by the multifunctional superhydrophobic fabrics are practically important because of the low cost and bio-degradable. The fibers with porous structure also possess excellent recyclability, ease in control of liquid penetration and motion, and can be self-cleaning or resist contamination by the various kinds of liquids or solvents. These smart ways of producing superhydrophobic fabrics result in highly stable surface properties under a wide range of conditions, such as the resistance of droplet sticking, high temperature, UV light, and high-concentration acidic/base solutions. 

Superhydrophobic fabrics with multifunctional applications have attracted great attention, and great progress has been made in recent years. However, up to now, most super-antiwetting fabrics are constructed by multiple steps/processes, which require precious equipment and/or harsh conditions to control the surface properties. Many problems still exist, such as mechanical stability and cost of materials with multi-functionalities, and need to be addressed before scalable production can be realized. Therefore, flexible, renewable and smart responsive fabrics with robust super-antiwetting property is a key issue for future investigation. Moreover, the air-permeation and fast adhesive force measurement on the superhydrophobic still needs to be improved.

As we all know, creatures and plants in Nature possess interesting and mysterious properties that we do not yet know. Therefore, further exploration and explanation of surfaces with special wetting behavior in nature is necessary. Learning from Nature will give us inspiration to develop simple and low-cost methods to construct artificial functional surfaces with superwettability for promising advanced applications [227,228,229,230,231]. We expect that more scientists interest and involve in these fields and further discover the relationship between chemical components and superwettability. Meanwhile, it has been recognized that some fluorochemicals and organic solvents have potential risks to human health and the environment and their use should be minimized. Environmental issues should be taken into account when preparing superhydrophobic surfaces for everyday use. 

## Figures and Tables

**Table 1:** Common techniques to construct superhydrophobic coatings on cellulose-based substrates and general characteristics of the different techniques and coatings.

Methods	Roughness Formation	Time-Scale and Requirement ^a^	Properties	Ref.
Dip-coating	Nanoparticle coating	Slow	Mechanical and environmental stability	[51,52,53,54,55,56,57,58,59,60,61,62,63]
Wet chemical etching	Growth of nano-structures by etching	Rapid/slow	Excellent resistance to washing, abrasion	[64,65,66,67]
Chemical bath disposition	Nanoparticle film deposition	Slow and temperature requirement	Moderate durability	[68,69,70,71,72,73,74,75]
Electrophoretic deposition	Nanoparticle coating	Rapid and conductive substrate requirement	Chemical stability, highly transparent	[76,77,78,79,80]
Electrospinning	Nanofibers by electrospinning	Slow and solvent requirement	Porous membrane	[81,82,83,84,85,86,87]
Spray-coating methods	Micro/nanostructures by spraying	Rapid and scalable under ambient conditions	Moderate stability, easy reparability	[88,89,90,91,92,93,94,95]
Chemical vapor deposition	Growth of nano structures by polymerization	Slow and need heating	Separation of oils or organic contaminates from water	[96,97,98,99,100,101]
Plasma etching process	Growth of nanostructures by etching	Moderate and require specific equipment	Self-cleaning	[102,103,104,105,106,107,108,109,110]

^a^ Typical time-scales for slow, moderate, and rapid procedures are 1 h or more, 1–5 min, and <30 s, respectively.

## References

[1] Wang R., Hashimoto K., Fujishima A., Chikuni M., Kojima E., Kitamura A., Shimohigoshi M., Watanabe T. (1997). Light-induced amphiphilic surfaces. Nature.

[2] Feng L., Li S.H., Li Y.S., Li H.J., Zhang L.J., Zhai J., Song Y.L., Liu B.Q., Jiang L., Zhu D.B. (2002). Super-hydrophobic surfaces: From natural to artificial. Adv. Mater..

[3] Sun T.L., Feng L., Gao X.F., Jiang L. (2005). Bioinspired surfaces with special wettability. Acc. Chem. Res..

[4] Wang S.T., Liu K.S., Yao X., Jiang L. (2015). Bioinspired surfaces with superwettability: New insight on theory, design, and applications. Chem. Rev..

[5] Oberli L., Caruso D., Hall C., Fabretto M., Murphy P.J., Evans D. (2014). Condensation and freezing of droplets on superhydrophobic surfaces. Adv. Colloid Interfaces Sci..

[6] Lafuma A., Quéré D. (2003). Superhydrophobic states. Nat. Mater..

[7] Xia F., Jiang L. (2008). Bio-inspired, smart, multiscale interfacial materials. Adv. Mater..

[8] Liu M.J., Zheng Y.M., Zhai J., Jiang L. (2010). Bioinspired super-antiwetting interfaces with special liquid-solid adhesion. Acc. Chem. Res..

[9] Darmanin T., Guittard F. (2014). Recent advances in the potential applications of bioinspired superhydrophobic materials. J. Mater. Chem. A.

[10] Li X.M., Reinhoudt D., Crego-Calama M. (2007). What do we need for a superhydrophobic surface? A review on the recent progress in the preparation of superhydrophobic surfaces. Chem. Soc. Rev..

[11] Bhushan B., Jung Y.C. (2011). Natural and biomimetic artificial surfaces for superhydrophobicity, self-cleaning, low adhesion, and drag reduction. Prog. Mater. Sci..

[12] Jiang T., Guo Z.G., Liu W.M. (2015). Biomimetic superoleophobic surfaces: Focusing on their fabrication and applications. J. Mater. Chem. A.

[13] Liu K.S., Cao M.Y., Fujishima A., Jiang L. (2014). Bio-inspired titanium dioxide materials with special wettability and their applications. Chem. Rev..

[14] Nagappan S., Ha C.S. (2015). Emerging trends in superhydrophobic surface based magnetic materials: Fabrications and their potential applications. J. Mater. Chem. A.

[15] Sahoo B.N., Kandasubramanian B. (2014). Recent progress in fabrication and characterisation of hierarchical biomimetic superhydrophobic structures. RSC Adv..

[16] Furstner R., Barthlott W., Neinhuis C., Wallzel P. (2005). Wetting and self-cleaning properties of artificial superhydrophobic surfaces. Langmuir.

[17] Marmur A. (2004). The lotus effect: Superhydrophobicity and metastability. Langmuir.

[18] Gao L.C., McCarthy T.J. (2006). The “lotus effect” explained: Two reasons why two length scales of topography are important. Langmuir.

[19] Wenzel R.N. (1936). Resistance of solid surfaces to wetting by water. Ind. Eng. Chem..

[20] Cassie A.B.D., Baxter S. (1944). Wettability of porous surfaces. Trans. Faraday Soc..

[21] Wang S., Jiang L. (2007). Definition of superhydrophobic states. Adv. Mater..

[22] Barthlott W., Neinhuis C. (1997). Purity of the sacred lotus, or escape from contamination in biological surfaces. Planta.

[23] Feng L., Zhang Y., Xi J., Zhu Y., Wang N., Xia F., Jiang L. (2008). Petal effect:  A superhydrophobic state with high adhesive force. Langmuir.

[24] Zheng Y.M., Bai H., Huang Z.B., Tian X.L., Nie F.Q., Zhao Y., Zhai J., Jiang L. (2010). Directional water collection on wetted spider silk. Nature.

[25] Parker A.R., Lawrence C.R. (2001). Water capture by a desert beetle. Nature.

[26] Gao X.F., Jiang L. (2004). Biophysics: Water-repellent legs of water striders. Nature.

[27] Lee H., Lee B.P., Messersmith P.B. (2007). A reversible wet/dry adhesive inspired by mussels and geckos. Nature.

[28] Zhang J.P., Wu L., Zhang Y.J., Wang A.Q. (2015). Mussel and fish scale-inspired underwater superoleophobic kapok membranes for continuous and simultaneous removal of insoluble oils and soluble dyes in water. J. Mater. Chem. A.

[29] Teisala H., Tuominen M., Kuusipalo J. (2014). Superhydrophobic coatings on cellulose-based materials: Fabrication, properties, and applications. Adv. Mater. Interfaces.

[30] Lai Y.K., Chen Z., Lin C.J. (2011). Recent progress on the superhydrophobic surfaces with special adhesion: From natural to biomimetic to functional. J. Nanoeng. Nanomanuf..

[31] Hejazi I., Sadeghi G.M.M., Jafari S.H., Khonakdar H.A., Seyfi J., Holzschuh M., Simon F. (2015). Transforming an intrinsically hydrophilic polymer to a robust self-cleaning superhydrophobic coating via carbon nanotube surface embedding. Mater. Design.

[32] Nguyen S.H., Webb H.K., Mahon P.J., Crawford R.J., Ivanova E.P. (2014). Natural insect and plant micro-/nanostructsured surfaces: An excellent selection of valuable templates with superhydrophobic and self-cleaning properties. Molecules.

[33] Erbil H.Y., Demirel A.L., Avci Y., Mert O. (2003). Transformation of a simple plastic into a superhydrophobic surface. Science.

[34] Xiao M., Guo X.P., Cheng M.J., Ju G.N., Zhang Y.J., Shi F. (2014). pH-responsive on-off motion of a superhydrophobic boat: Towards the design of a minirobot. Small.

[35] Cheng M.J., Liu Q., Ju G.N., Zhang Y.J., Jiang L., Shi F. (2014). Superhydrophilic-superhydrophobic-superhydrophilic double transformation on a pH-responsive smart surface. Adv. Mater..

[36] Lai Y.K., Lin C.J., Huang J.Y., Zhuang H.F., Sun L., Nguyen T. (2008). Markedly controllable adhesion of superhydrophobic sponge-like nanostructure TiO_2_ films. Langmuir.

[37] Celia E., Darmanin T., de Givenchy E.T., Amigoni S., Guittard F. (2013). Recent advances in designing superhydrophobic surfaces. J. Colloid. Interface Sci..

[38] Kumar D., Wu X.H., Fu Q.T., Ho J.W.C., Kanhere P.D., Li L., Chen Z. (2015). Hydrophobic sol-gel coatings based on polydimethylsiloxane for self-cleaning applications. Mater. Design.

[39] Lai Y.K., Huang J.Y., Cui Z.Q., Ge M.Z., Zhang K.Q., Chen Z., Chi L.F. (2016). Recent advances in TiO_2_-based nanostructure surfaces with controllable wettability and adhesion. Small.

[40] Teng Y.Q., Zhang Y.Q., Heng L.P., Meng X.F., Yang Q.W., Jiang L. (2015). Conductive polymer porous film with tunable wettability and adhesion. Materials.

[41] Lai Y.K., Chen Y.C., Tang Y.X., Gong D.G., Chen Z., Lin C.J. (2009). Electrophoretic deposition of titanate nanotube films with extremely large wetting contrast. Electrochem. Commun..

[42] Cheng M.J., Song M.M., Dong H.Y., Shi F. (2015). Surface adhesive forces: A metric describing the drag-reducing effects of superhydrophobic coatings. Small.

[43] Huan S.Q., Liu G.X., Han G.P., Cheng W.L., Fu Z.Y., Wu Q.L., Wang Q.W. (2015). Effect of experimental parameters on morphological, mechanical and hydrophobic properties of electrospun polystyrene fibers. Materials.

[44] Wang B., Zhang Y.B., Liang W.X., Wang G.Y., Guo Z.G., Liu W.M. (2015). A simple route to transform normal hydrophilic cloth into a superhydrophobic-superhydrophilic hybrid surface. J. Mater. Chem. A.

[45] Huang J.Y., Lai Y.K., Wang L.N., Li S.H., Ge M.Z., Zhang K.Q., Fuchs H., Chi L.F. (2014). Controllable wettability and adhesion on bioinspired multifunctional TiO_2_ nanostructure surfaces for liquid manipulation. J. Mater. Chem. A.

[46] Lai Y.K., Tang Y.X., Huang J.Y., Wang H., Li H.Q., Gong D.G., Ji X.B., Gong J.J., Lin C.J., Sun L. (2011). Multi-functional hybrid protonated titanate nanobelts with tunable wettability. Soft Matter.

[47] Ma M.L., Mao Y., Gupta M., Gleason K.K., Rutledge G.C. (2005). Superhydrophobic fabrics produced by electrospinning and chemical vapor deposition. Macromolecules.

[48] Tian D.L., Zhang X.F., Wang X., Zhai J., Jiang L. (2011). Micro/nanoscale hierarchical structured ZnO mesh film for separation of water and oil. Phys. Chem. Chem. Phys..

[49] Pan S.J., Kota A.K., Mabry J.M., Tuteja A. (2013). Superomniphobic surfaces for effective chemical shielding. J. Am. Chem. Soc..

[50] Darmanin T., Tarrade J., Celia E., Bellanger H., Guittard F. (2014). Superoleophobic meshes with relatively low hysteresis and sliding angles by electropolymerization: Importance of polymer-growth control. ChemPlusChem.

[51] Zhang X., Geng T., Guo Y.G., Zhang Z.J., Zhang P.Y. (2013). Facile fabrication of stable superhydrophobic SiO_2_/polystyrene coating and separation of liquids with different surface tention. Chem. Eng. J..

[52] Hu H.B., Gao L., Chen C.L., Chen Q.W. (2014). Low-cost, acid/alkaline-resistant, and fluorine-free superhydrophobic fabric coating from onionlike carbon microspheres converted from waste polyethylene terephthalate. Environ. Sci. Technol..

[53] Cengiz U., Erbil H.Y. (2014). Superhydrophobic perfluoropolymer surfaces having heterogeneous roughness created by dip-coating from solutions containing a nonsolvent. Appl. Surf. Sci..

[54] Lin J., Zheng C., Ye W.J., Wang H.Q., Feng D.Y., Li Q.Y., Huan B.W. (2015). A facile dip-coating approach to prepare SiO_2_/fluoropolymer coating for superhydrophobic and superoleophobic fabrics with self-cleaning property. J. Appl. Polym. Sci..

[55] Makowski T., Kowalczyk D., Fortuniak W., Jeziorska D., Brzezinski S., Tracz A. (2014). Superhydrophobic properties of cotton woven fabrics with conducting 3D networks of multiwall carbon nanotubes, MWCNTs. Cellulose.

[56] Wang J.T., Chen Y.H. (2015). Oil-water separation capability of superhydrophobic fabrics fabricated via combining polydopamine adhesion with lotus-leaf-like structure. J. Appl. Polym. Sci..

[57] Nateghi M.R., Shateri-Khalilabad M.R. (2015). Silver nanowire-functionalized cotton fabric. J. Carbohyd. Polym..

[58] Bayer I.S., Fragouli D., Attanasio A., Sorce B., Bertoni G., Brescia R., di Corato R., Pellegrino T., Kalyva M., Sabella S. (2011). Water-repellent cellulose fiber networks with multifunctional properties. ACS Appl. Mater. Interfaces.

[59] Wang S.H., Li M., Lu Q.H. (2010). Filter paper with selective absorption and separation of liquids that differ in surface tension. ACS Appl. Mater. Interfaces.

[60] Li S.H., Zhang S.B., Wang X.H. (2008). Fabrication of superhydrophobic cellulose-based materials through a solution-immersion process. Langmuir.

[61] Zhou H., Wang H.X., Niu H.T., Gestos A., Wang X.G., Lin T. (2012). Fluoroalkyl silane modified silicone rubber/nanoparticle composite: A super durable, robust superhydrophobic fabric coating. Adv. Mater..

[62] Zhou H., Wang H.X., Niu H.T., Gestos A., Lin T. (2013). Robust, self-healing superamphiphobic fabrics prepared by two-step coating of fluoro-containing polymer, fluoroalkyl silane, and modified silica nanoparticles. Adv. Funct. Mater..

[63] Wang H.X., Fang J., Cheng T., Ding J., Qu L.T., Dai L.M., Wang X.G., Lin T. (2008). One-step coating of fluoro-containing silica nanoparticles for universal generation of surface sueperhydrophobicity. Chem. Commun..

[64] Wu L., Zhang J., Li B., Fan L., Li L., Wang A. (2014). Facile preparation of super durable superhydrophobic materials. J. Colloid. Interface Sci..

[65] Gupta N., Sasikala S., Barshilia H.C. (2012). Corrosion study of superhydrophobic magnesium alloy AZ31 surfaces prepared by wet chemical etching process. Nanosci. Nanotech. Lett..

[66] Lai Y.K., Gao X.F., Zhuang H.F., Huang J.Y., Lin C.J., Jiang L. (2009). Designing superhydrophobic porous nanostructures with tunable water adhesion. Adv. Mater..

[67] Lai Y.K., Sun L., Chen Y.C., Zhuang H.F., Lin C.J., Chin J.W. (2006). Effects of the structure of TiO_2_ nanotube array on Ti substrate on its photocatalytic activity. J. Electrochem. Soc..

[68] Xue C.H., Li Y.R., Zhang P., Ma J.Z., Jia S.T. (2014). Washable and wear-resistant superhydrophobic surfaces with self-cleaning property by chemical etching of fibers and hydrophobization. ACS Appl. Mater. Interfaces.

[69] Zeng J.W., Wang B., Zhang Y.B., Zhu H., Guo Z.G. (2014). Strong amphiphobic porous films with oily-self-cleaning property beyond nature. Chem. Lett..

[70] Huang J.Y., Li S.H., Ge M.Z., Wang L.N., Xing T.L., Chen G.Q., Liu X.F., Al-Deyab S.S., Zhang K.Q., Chen T. (2015). Robust superhydrophobic TiO_2_@fabrics for UV shielding, self-cleaning and oil-water separation. J. Mater. Chem. A.

[71] Zhang M., Zang D.L., Shi J.Y., Gao Z.X., Wang C.Y., Li J. (2015). Superhydrophobic cotton textile with robust composite film and flame retardancy. RSC Adv..

[72] Kivotidi S., Tsioptsias C., Pavlidou E., Panayiotou C. (2012). Flame-retarded hydrophobic cellulose through impregnation with aqueous solutions and supercritical CO_2_. J. Therm. Anal. Calorim..

[73] Xu B., Cai Z.S. (2008). Fabrication of a superhydrophobic ZnO nanorod array film on cotton fabrics via a wet chemical route and hydrophobic modification. Appl. Surf. Sci..

[74] Li S.H., Huang J.Y., Ge M.Z., Cao C.Y., Deng S., Zhang S.N., Chen G.Q., Zhang K.Q., Al-Deyab S.S., Lai Y.K. (2015). Robust flower-like TiO_2_@Cotton fabrics with special wettability for effective self-cleaning and versatile oil/water separation. Adv. Mater. Interfaces.

[75] Zhang T., Yan H.Q., Fang Z.P., E Y.P., Wu T., Chen F. (2014). Superhydrophobic and conductive properties of carbon nanotubes/polybenzoxazine nanocomposites coated ramie fabric prepared by solution-immersion process. Appl. Surf. Sci..

[76] Lai Y.K., Tang Y.X., Gong J.J., Gong D.G., Chi L.F., Lin C.J., Chen Z. (2012). Transparent superhydrophobic/superhydrophilic TiO_2_-based coatings for self-cleaning and anti-fogging. J. Mater. Chem..

[77] Joung Y.S., Buie C.R. (2015). Anti-wetting fabric produced by a combination of layer-by-layer assembly and electrophoretic deposition of hydrophobic nanoparticles. ACS Appl. Mater. Interfaces.

[78] Huang Y., Sarkar D.K., Chen X.G. (2015). Superhydrophobic nanostructured ZnO thin films on aluminum alloy substrates by electrophoretic deposition process. Appl. Surf. Sci..

[79] Ogihara H., Katayama T., Saji T. (2011). One-step electrophoretic deposition for the preparation of superhydrophobic silica particle/trimethylsiloxysilicate composite coatings. J. Colloid Interface Sci..

[80] Zhao N., Shi F., Wang Z.Q., Zhang X. (2005). Combining layer-by-layer assembly with electrodeposition of silver aggregates for fabricating superhydrophobic surfaces. Langmuir.

[81] Oktay B., Toker R.D., Kayaman-Apohan N. (2015). Superhydrophobic behavior of polyimide-siloxane mats produced by electrospinning. Polym. Bull..

[82] Cakir M., Kartal I., Yildiz Z. (2014). The preparation of UV-cured superhydrophobic cotton fabric surfaces by electrospinning method. Textile Res. J..

[83] Han D.W., Steckl A.J. (2009). Superhydrophobic and oleophobic fibers by coaxial electrospinning. Langmuir.

[84] Zhou Z.P., Wu X.F. (2015). Electrospinning superhydrophobic-superoleophilic fibrous PVDF membranes for high-efficiency water-oil separation. Mater. Lett..

[85] Wang X.F., Ding B., Yu J.Y., Wang M.R. (2011). Engineering biomimetic superhydrophobic surfaces of electrospun nanomaterials. Nano Today.

[86] Zhu M.F., Zuo W.W., Yu H., Yang W., Chen Y.M. (2006). Superhydrophobic surface directly created by electrospinning based on hydrophilic material. J. Mater. Sci..

[87] Nuraje N., Khan W.S., Lei Y., Ceylan M., Asmatulu R. (2013). Superhydrophobic electrospun nanofibers. J. Mater. Chem. A.

[88] Spark B.J., Hoff E.F.T., Xiong L., Goetz J.T., Patton D.L. (2013). Superhydrophobic hybrid inorganic-organic thiol-ene surfaces fabricated via spray-deposition and photopolymerization. ACS Appl. Mater. Interfaces.

[89] Yang J., Tang Y.C., Xu J.Q., Chen B.B., Tang H., Li C.S. (2015). Durable superhydrophobic/superoleophilic epoxy/attapulgite nanocomposite coatings for oil/water separation. Surf. Coat. Technol..

[90] Ge D.T., Yang L.L., Wu G.X., Yang S. (2014). Spray coating of superhydrophobic and angle-independent coloured films. Chem. Commun..

[91] Li B.C., Zhang J.P., Wu L., Wang A.Q. (2013). Durable superhydrophobic surfaces prepared by spray coating of polymerized organosilane/attapulgite nanocomposites. ChemPlusChem.

[92] Chu Z.L., Seeger S. (2015). Robust superhydrophobic wood obtained by spraying silicone nanoparticles. RSC Adv..

[93] Li J., Jing Z.J., Zha F., Yang Y.X., Wang Q.T., Lei Z.Q. (2014). Facile spray-coating process for the fabrication of tunable adhesive superhydrophobic surfaces with heterogeneous chemical compositions used for selective transportation of microdroplets with different volumes. ACS Appl. Mater. Interfaces.

[94] Ma J.Z., Zhang X.Y., Bao Y., Liu J.L. (2015). A facile spraying method for fabricating superhydrophobic leather coating. Colloids Surf. A.

[95] Kim D.Y., Lee J.G., Joshi B.N., Latthe S.S., Al-Deyab S.S., Yoon S.S. (2015). Self-cleaning superhydrophobic films by supersonic-spraying polytetrafluoroethylene-titania nanoparticles. J. Mater. Chem. A.

[96] Artus C.R.J., Jung S., Zimmermann J., Gautschi H.P., Marquardt K., Seeger S. (2006). Silicone nanofilaments and their application as superhydrophobic coatings. Adv. Mater..

[97] Balu B., Breedveld V., Hess D.W. (2008). Fabrication of “roll-off” and “sticky” superhydrophobic cellulose surfaces via plasma processing. Langmuir.

[98] Bao X.M., Cui J.F., Sun H.X., Liang W.D., Zhu Z.Q., An J., Yang B.P., La P.Q., Li A. (2014). Facile preparation of superhydrophobic surfaces based on metal oxide nanoparticles. Appl. Surf. Sci..

[99] Zhou X.Y., Zhang Z.Z. (2013). Robust and durable superhydrophobic cotton fabrics for oil/water separation. ACS Appl. Mater. Interfaces.

[100] Aminayi P., Abidi N. (2015). Ultra-oleophobic cotton fabric prepared using molecular and nanoparticle vapor deposition methods. Surf. Coat. Technol..

[101] Aminayi P., Abidi N. (2013). Imparting super hydro/oleophobic properties to cotton fabric by means of molecular and nanoparticles vapor deposition methods. Appl. Surf. Sci..

[102] Oh J., Ko T., Moon M., Park C.H. (2014). Nanostructured superhydrophobic silk fabric fabricated using the ion beam method. RSC Adv..

[103] Zheng Z.R., Gu Z.Y., Huo R.T., Ye Y.H. (2009). Superhydrophobicity of polyvinylidene fluoride membrane fabricated by chemical vapor deposition from solution. Appl. Surf. Sci..

[104] Chakraborty A., Xiang M.M., Luo C. (2013). Fabrication of super-hydrophobic microchannels via strain-recovery deformations of polystyrene and oxygen reactive ion etch. Materials.

[105] Caschera D., Mezzi A., Cerri L., de Caro T., Riccucci C., Ingo G.M., Padeletti G., Biasiucci M., Gigli G., Cortese B. (2014). Effects of plasma treatments for improving extreme wettability behavior of cotton fabrics. Cellulose.

[106] Caschera D., Toro R.G., Federici F., Riccucci C., Ingo G.M., Gigli G., Cortese B. (2015). Flame retardant properties of plasma pre-treated/diamond-like carbon (DLC) coated cotton fabrics. Cellulose.

[107] Kwon S.O., Ko T.J., Yu E.S., Kim J., Moon M.W., Park C.H. (2014). Nanostructured self-cleaning lyocell fabrics with asymmetric wettability and moisture absorbency (part I). RSC Adv..

[108] Kinoshita H., Ogasahara A., Fukuda Y., Ohmae N. (2010). Superhydrophobic/superhydrophilic micropatterning on a carbon nanotube film using a laser plasma-type hyperthermal atom beam facility. Carbon.

[109] Yang C., Li X.M., Gilron J., Kong D.F., Yin Y., Oren Y., Linder C., He T. (2014). CF_4_ plasma-modified superhydrophobic PVDF membranes for direct contact membrane distillation. J. Membrane Sci..

[110] Ellinas K., Pujari S.P., Dragatogiannis D.A., Charitidis C.A., Tserepi A., Zuilhof H., Gogolides E. (2014). Plasma micro-nanotextured, scratch, water and hexadecane resistant, superhydrophobic, and superamphiphobic polymeric surfaces with perfluorinated monolayers. ACS Appl. Mater. Interfaces.

[111] Li S.H., Huang J.Y., Ge M.Z., Li S.W., Xing T.L., Chen G.Q., Liu Y.Q., Zhang K.Q., Al-Deyab S.S., Lai Y.K. (2015). Controlled grafting superhydrophobic cellulose surface with environmentally-friendly short fluoroalkyl chains by ATRP. Mater. Design.

[112] Zhang Y., Li Y.Q., Shao J.Z., Zou C. (2015). Fabrication of superhydrophobic fluorine-free films on cotton fabrics through plasma-induced grafting polymerization of 1,3,5,7-tetravinyl-1,3,5,7-tetramethyl cyclotetrasiloxane. Surf. Coat. Technol..

[113] Li Y.W., Zheng X.W., Zhu H.Y., Wu K., Lu M.G. (2015). Synthesis and self-assembly of well-defined binary graft copolymer and its use in superhydrophobic cotton fabrics preparation. RSC Adv..

[114] Duan W., Xie A.J., Shen Y.H., Wang Y.F., Wang F., Zhang Y., Li J.L. (2011). Fabrication of superhydrophobic cotton fabrics with UV protection based on CeO_2_ particles. Ind. Eng. Chem. Res..

[115] Wang H.X., Ding J., Xue Y.H., Wang X.G., Lin T. (2010). Superhydrophobic fabrics from hybrid silica sol-gel coatings: Structural effect of precursors on wettability and washing durability. J. Mater. Res..

[116] Gao Q.W., Zhu Q., Guo Y.L., Yang C.Q. (2009). Formation of highly hydrophobic surfaces on cotton and polyester fabrics using silica sol nanoparticles and nonfluorinated alkylsilane. Ind. Eng. Chem. Res..

[117] Gurav A.B., Xu Q.F., Latthe S.S., Vhatkar R.S., Liu S.H., Yoon H., Yoon S.S. (2015). Superhydrophobic coatings prepared from methyl-modified silica particles using simple dip-coating method. Ceram. Int..

[118] Zhu T., Cai C., Duan C.T., Zhai S., Liang S.M., Jin Y., Zhao N., Xu J. (2015). Robust polypropylene fabrics super-repelling various liquids: A simple, rapid and scalable fabrication method by solvent swelling. ACS Appl. Mater. Interfaces.

[119] Chen F.X., Liu X., Yang H.Y., Dong B.H., Zhou Y.S., Chen D.Z., Hu H., Xiao X.F., Fan D.F., Zhang C.H. (2016). A simple one-step approach to fabrication of highly hydrophobic silk fabrics. Appl. Surf. Sci..

[120] Xiao X.F., Cao G.Y., Chen F.X., Tang Y.R., Liu X., Xu W.L. (2015). Durable superhydrophobic wool fabrics coating with nanoscale Al_2_O_3_ layer by atomic layer deposition. Appl. Surf. Sci..

[121] Cortese B., Caschera D., Federici F., Ingo G.M., Gigli G. (2014). Superhydrophobic fabrics for oil-water separation through a diamond like carbon (DLC) coating. J. Mater. Chem. A.

[122] Wang F.J., Yu S., Ou J.F., Xue M.S., Li W. (2013). Mechanically durable superhydrophobic surfaces prepared by abrading. J. Appl. Phys..

[123] Yu M., Wang Z.Q., Liu H.Z., Xie S.Y., Wu J.X., Jiang H.Q., Zhang J.Y., Li L.F., Li J.Y. (2013). Laundering durability of photocatalyzed self-cleaning cotton fabric with TiO_2_ nanoparticles covalently immobilized. ACS Appl. Mater. Interfaces.

[124] Wu B.T., Zhang B.W., Wu J.X., Wang Z.Q., Ma H.J., Yu M., Li L.F., Li J.Y. (2015). Electrical switchability and dry-wash durability of conductive textiles. Sci. Rep..

[125] Wang B., Li J., Wang G.Y., Liang W.X., Zhang Y.B., Shi L., Guo Z.G., Liu W.M. (2013). Methodology for robust superhydrophobic fabrics and sponges from *in situ* growth of transition metal/metal oxide nanocrystals with thiol modification and their applications in oil/water separation. ACS Appl. Mater. Interfaces.

[126] Guo Z.G., Zhou F., Hao J.C., Liu W.M. (2005). Stable biomimetic super-hydrophobic engineering materials. J. Am. Chem. Soc..

[127] Wang B., Liang W.X., Guo Z.G., Liu W.M. (2015). Biomimetic super-lyophobic and super-lyophilic materials applied for oil/water separation: a new strategy beyond nature. Chem. Soc. Rev..

[128] Liu F., Ma M.L., Zang D.L., Gao Z.X., Wang C.Y. (2014). Fabrication of superhydrophobic/superoleophilic cotton for application in the field of water/oil separation. Carbohyd. Poly..

[129] Huang S.Y. (2014). Mussel-inspired one-step copolymerization to engineer hierarchically structured surface with superhydrophobic properties for removing oil from water. ACS Appl. Mater. Inter.

[130] Wang Z.X., Xu Y.C., Liu Y.Y., Shao L.J. (2015). A novel mussel-inspired strategy toward superhydrophobic surfaces for self-driven crude oil spill cleanup. J. Mater. Chem. A.

[131] Wang Y.C., Tao S.Y., An Y.G. (2013). A reverse membrane emulsification process based on a hierarchically porous monolith for high efficiency water-oil separation. J. Mater. Chem. A.

[132] Zhu X.T., Zhang Z.Z., Ge B., Men X.H., Zhou X.Y., Xue Q.J. (2014). A versatile approach to produce superhydrophobic materials used for oil-water separation. J. Colloid. Interface Sci..

[133] Zhang W.F., Lu X., Xin Z., Zhou C.L. (2015). A self-cleaning polybenzoxazine/TiO_2_ surface with superhydrophobicity and superoleophilicity for oil/water separation. Nanoscale.

[134] Liang J., Zhou Y., Jiang G.H., Wang R.J., Wang X.H., Hu R.B., Xi X.G. (2013). Transformation of hydrophilic cotton fabrics into superhydrophobic surfaces for oil/water separation. J. Text. Inst..

[135] Song J., Huang S., Lu Y., Bu X., Mates J.E., Ghosh A., Ganguly R., Carmalt C.J., Parkin I.P., Xu W. (2014). Self-driven one-step oil removal from oil spill on water via selective-wettability steel mesh. ACS Appl. Mater. Interfaces.

[136] Zang D.L., Liu F., Zhang M., Niu X.G., Gao Z.X., Wang C.Y. (2015). Superhydrophobic coating on fiberglass cloth for selective removal of oil from water. Chem. Eng. J..

[137] Li J., Yan L., Zhao Y.Z., Zha F., Wang Q.T., Lei Z.Q. (2015). Correction: One-step fabrication of robust fabrics with both-faced superhydrophobicity for the separation and capture of oil from water. Phys. Chem. Chem. Phys..

[138] Dudchenko A.V., Rolf J., Shi L., Olivas L., Duan W.Y., Jassby D. (2015). Coupling underwater superoleophobic membranes with magnetic pickering emulsions for fouling-free separation of crude oil/water mixtures: An experimental and theoretical study. ACS Nano.

[139] Li K.Q., Zeng X.R., Li H.Q., Lai X.J., Xie H. (2014). Facile fabrication of superhydrophobic filtration fabric with honeycomb structures for the separation of water and oil. Mater. Lett..

[140] Arumugham T., Kaleekkal N.J., Rana D., Doraiswamy M. (2016). Separation of oil/water emulsions using nano MgO anchored hybrid ultrafiltration membranes for environmental abatement. J. Appl. Poly. Sci..

[141] Huang M.L., Si Y., Tang X.M., Zhu Z.G., Ding B., Liu L.F., Zheng G., Luo W.J., Yu J.Y. (2013). Gravity driven separation of emulsified oil-water mixtures utilizing *in situ* polymerized superhydrophobic and superoleophilic nanofibrous membranes. J. Mater. Chem. A.

[142] Si Y., Fu Q., Wang X., Zhu J., Yu J., Sun G., Ding B. (2015). Superelastic and superhydrophobic nanofiber-assembled cellular aerogels for effective separation of oil/water emulsions. ACS Nano.

[143] Si Y., Yan CC., Hong F.F., Yu J.Y., Ding B. (2015). A general strategy for fabricating flexible magnetic silica nanofibrous membranes with multifunctionality. Chem. Commun..

[144] Zhang L.B., Zhang Z.H., Wang P. (2012). Smart surfaces with switchable superoleophilicity and superoleophobicity in aqueous media: toward controllable oil/water separation. NPG Asia Mater..

[145] Jin Y.X., Ke Q.P., Jiang P., Zhu Y.S.N., Cheng F.H., Zhang Y.X. (2015). High efficiently oil/water separation and excellent self-cleaning surfaces based on 1-triacontanol-polymerized octadecylsiloxane coatings. Appl. Surf. Sci..

[146] Poortavasoly H., Montazer M., Harifi T. (2016). Aminolysis of polyethylene terephthalate surface along with *in situ* synthesis and stabilizing ZnO nanoparticles using triethanolamine optimized with response surface methodology. Mater. Sci. Eng. C.

[147] Mura S., Greppi G., Malfatti L., Lasio B., Sanna V., Mura M.E., Marceddu S., Lugliè L. (2015). Multifunctionalization of wool fabrics through nanoparticles: A chemical route towards smart textiles. J. Colloid Interface Sci..

[148] Banerjee S., Dionysiou D.D., Pillai S.C. (2015). Self-cleaning applications of TiO_2_ by photo-induced hydrophilicity and photocatalysis. Appl. Catal. B.

[149] Shahidi S., Ahmadi M., Rashidi A., Ghoranneviss M. (2015). Effect of plasma treatment on self-cleaning of textile fabric using titanium dioxide. Micro Nano Lett..

[150] Manna J., Goswami S., Shilpa N., Sahu N., Rana R.K. (2015). Biomimetic method to assemble nanostructured Ag@ZnO on cotton fabrics: Application as self-cleaning flexible materials with visible-light photocatalysis and antibacterial activities. ACS Appl. Mater. Interfaces.

[151] Ge M.Z., Cao C.Y., Huang J.Y., Li S.H., Chen Z., Zhang K.Q., Al-Deyab S.S., Lai Y.K. (2016). A review of one-dimensional TiO_2_ nanostructured materials for environmental and energy applications. J. Mater. Chem. A.

[152] Xu B., Ding J., Feng L., Ding Y.Y., Ge F.Y., Cai Z.S. (2015). Self-cleaning cotton fabrics via combination of photocatalytic TiO_2_ and superhydrophobic SiO_2_. Surf. Coat. Technol..

[153] Zheng X., Guo Z.Y., Tian D.L., Zhang X.F., Li W.X., Jiang L. (2015). Underwater self-cleaning scaly fabric membrane for oily water separation. ACS Appl. Mater. Interfaces.

[154] Li J.H., Park E.J., Kim D.H., Jeong M.G., Kim Y.D. (2016). Superhydrophobic surfaces with photocatalytic activity under UV and visible light irradiation. Catal. Today.

[155] Afzal S., Daoud W.A., Langford S.J. (2014). Superhydrophobic and photocatalytic self-cleaning cotton. J. Mater. Chem. A.

[156] Khajavi R., Berendjchi A. (2014). Effect of dicarboxylic acid chain length on the self-cleaning property of nano-TiO_2_-coated cotton fabrics. ACS Appl. Mater. Interfaces.

[157] Behzadnia A., Montazer M., Rashidi A., Rad M.M. (2014). Rapid sonosynthesis of N-doped nano TiO_2_ on wool fabric at low temperature: introducing self-cleaning, hydrophilicity, antibacterial/antifungal properties with low alkali solubility, yellowness and cytotoxicity. J. Photochem. Photobiol..

[158] Karimi L., Yazdanshenas M.E., Khajavi R., Rashidi A., Mirjalili M. (2014). Using graphene/TiO_2_ nanocomposite as a new route for preparation of electroconductive, self-cleaning, antibacterial and antifungal cotton fabric without toxicity. Cellulose.

[159] Teng C., Lu X.Y., Ren G.Y., Zhu Y., Wan M.X., Jiang L. (2014). Underwater self-cleaning PEDOT-PSS hydrogel mesh for effective separation of corrosive and hot oil/water mixtures. Adv. Mater. Interfaces.

[160] Zheng C.H., Qi Z.M., Shen W.C., Chen G.Q. (2014). Self-cleaning Bombyx mori silk: Room-temperature preparation of anatase nano-TiO_2_ by the sol-gel method and its application. Color. Technol..

[161] Ragesh P., Nair S.V., Nair A.S. (2014). An attempt to fabricate a photocatalytic and hydrophobic self-cleaning coating via electrospinning. RSC Adv..

[162] Barletta M., Vesco S., Tagliaferri V. (2014). *In situ* sonosynthesis of nano TiO_2_ on cotton fabric. J. Ultrason. Sonochem..

[163] Zohoori S., Karimi L., Nazari A. (2014). Photocatalytic self-cleaning synergism optimization of cotton fabric using nano SrTiO_3_ and nano TiO_2_. J. Fibres. Text. East. Eur..

[164] Xue C.H., Jia S.T., Zhang J., Ma J.Z. (2010). Large-area fabrication of superhydrophobic surfaces for practical applications: An overview. Sci. Technol. Adv. Mater..

[165] Barletta M., Vesco S., Tagliaferri V. (2014). Self-cleaning and self-sanitizing coatings on plastic fabrics: Design, manufacture and performance. J. Colloid Surface B.

[166] Zhao Y.Y., Liu Y., Xu Q.F., Barahman M., Lyons A.M. (2015). Catalytic, self-cleaning surface with stable superhydrophobic properties: Printed polydimethylsiloxane (PDMS) arrays embedded with TiO_2_ nanoparticles. ACS Appl. Mater. Interfaces.

[167] Ge M.Z., Cao C.Y., Huang J.Y., Li S.H., Zhang S.N., Deng S., Li Q.S., Zhang K.Q., Lai Y.K. (2016). Synthesis, modification, and photo/photoelectro catalytic degradation applications of TiO_2_ nanotube arrays: A review. Nanotechnol. Rev..

[168] Wang L., Xi G.H., Wan S.J., Zhao C.H., Liu X.D. (2014). Asymmetrically superhydrophobic cotton fabrics fabricated by mist polymerization of lauryl methacrylate. Cellulose.

[169] Xi G.H., Fan W.C., Wang L., Liu X.D., Endo T. (2015). Fabrication of asymmetrically superhydrophobic cotton fabrics via mist copolymerization of 2,2,2-trifluoroethyl methacrylate. J. Polym. Sci. Part A.

[170] Liu Y., Xin J.H., Choi C.H. (2012). Cotton fabrics with single-faced superhydrophobicity. Langmuir.

[171] Gu J.C., Xiao P., Chen J., Zhang J.W., Huang Y.J., Chen T. (2014). Janus polymer/carbon nanotube hybrid membranes for oil/water separation. ACS Appl. Mater. Interfaces.

[172] Wang H.X., Ding J., Dai L.M., Wang X.G., Lin T. (2010). Directional water-transfer through fabrics induced by asymmetric wettability. J. Mater. Chem..

[173] Liu Y., Wang X.W., Fei B., Hu H.W., Lai C.L., Xin J.H. (2015). Bioinspired, stimuli-responsive, multifunctional superhydrophobic surface with directional wetting, adhesion, and transport of water. Adv. Funct. Mater..

[174] Wang H.X., Zhou H., Yang W.D., Zhao Y., Fang J., Lin T. (2015). Selective, spontaneous one-way oil-transport fabrics and their novel use for gauging liquid surface tension. ACS Appl. Mater. Interfaces.

[175] Kong Y., Liu Y., Xin J.H. (2011). Fabrics with self-adaptive wettability controlled by “light-and-dark”. J. Mater. Chem..

[176] Zhou H., Wang H.X., Niu H.T., Lin T. (2013). Superphobicity/philicity Janus fabrics with switchable, spontaneous, directional transport ability to water and oil fluids. Sci. Rep..

[177] Lim H.S., Park S.H., Koo S.H., Kwark Y.J., Thomas E.L., Jeong Y.J., Cho J.H. (2010). Superamphiphilic Janus fabric. Langmuir.

[178] Wang H., Zhou H., Niu H., Zhang J., Du Y., Lin T. (2015). Dual-layer superamphiphobic/superhydrophobic-oleophilic nanofibrous membranes with unidirectional oil-transport ability and strengthened oil-water separation performance. Adv. Mater. Interfaces.

[179] Tian X.L., Jin H., Sainio J., Ras R.H.A., Ikkala O. (2014). Droplet and fluid gating by biomimetic Janus membranes. Adv. Funct. Mater..

[180] Xue C.H., Yin W. (2011). UV-durable superhydrophobic textiles with UV-shielding properties by coating fibers with ZnO/SiO_2_ core/shell particles. Nat Nanotechnol..

[181] Xue C.H., Yin W. (2013). UV-durable superhydrophobic textiles with UV-shielding properties by introduction of ZnO/SiO_2_ core/shell nanorods on PET fibers and hydrophobization. Colloid Surf. A..

[182] Wu J.X., Li J.Y., Wang Z.Q., Yu M., Jiang H.Q., Li L.F., Zhang B.W. (2015). Designing breathable superhydrophobic cotton fabrics. RSC Adv..

[183] Zeng C., Wang H.X., Zhou H., Lin T. (2015). Self-cleaning, superhydrophobic cotton fabrics with excellent washing durability, solvent resistance and chemical stability prepared from an SU-8 derived surface coating. RSC Adv..

[184] Brown P.S., Bhushan B. (2015). Mechanically durable, superoleophobic coatings prepared by layer-by-layer technique for anti-smudge and oil-water separation. Sci. Rep..

[185] Wang L.L., Zhang X.T. (2011). Superhydrophobic and ultraviolet-blocking cotton textiles. ACS Appl. Mater. Interfaces.

[186] Verho T., Bower C. (2011). Mechanically durable superhydrophobic surfaces. Adv. Mater..

[187] Liu J.Y., Huang W.Q. (2011). Preparation of durable superhydrophobic surface by sol-gel method with water glass and citric acid. Sol-Gel Sci. Technol..

[188] Zou L.H., Lan C.T. (2015). Superhydrophobization of cotton fabric with multiwalled carbon nanotubes for durable electromagnetic interference shielding. Fiber. Polym..

[189] Abbas R., Khereby M.A. (2015). Fabrication of durable and cost effective superhydrophobic cotton textiles via simple one step process. Cellulose.

[190] Zhou H., Wang H., Niu H., Fang J., Zhao Y., Lin T. (2015). Superstrong, chemically stable, superamphiphobic fabrics from particle-free polymer coatings. Adv. Mater. Interfaces.

[191] Tian D., Zhang X., Tian Y., Wu Y., Wang X., Zhai J., Jiang L. (2012). Photo-induced water-oil separation based on switchable superhydrophobicity-superhydrophilicity and underwater superoleophobicity of the aligned ZnO nanorod array-coated mesh films. J. Mater. Chem..

[192] Dunderdale G.J., Urata C., Miranda D.F., Hozumi A. (2014). Large-scale and environmentally friendly synthesis of pH-responsive oil-repellent polymer brush surfaces under ambient conditions. ACS Appl. Mater. Interfaces.

[193] Tsai Y.T., Choi C.H., Gao N., Yang E.H. (2011). Tunable wetting mechanism of polypyrrole surfaces and low-voltage droplet manipulation via redox. Langmuir.

[194] Liu Y.M., Sarshar M.A., Du K., Chou T., Choi C.H., Sukhishvili S.A. (2013). Large-amplitude, reversible, pH-triggered wetting transitions enabled by layer-by-layer films. ACS Appl. Mater. Interfaces.

[195] Kota A.K., Kwon G., Choi W., Mabry J.M., Tuteja A. (2012). Hygro-responsive membranes for effective oil-water separation. Nat. Commun..

[196] Wang B., Guo Z.G., Liu W.M. (2014). pH-responsive smart fabrics with controllable wettability in different surroundings. RSC Adv..

[197] Shateri-Khalilabad M., Yazdanshenas M.E. (2013). Preparation of superhydrophobic electroconductive graphene-coated cotton cellulose. Cellulose.

[198] Li M.M., Qing G.Y., Xiong Y.T., Lai Y.K., Sun T.L. (2015). CH-π interaction driven macroscopic property transition on smart polymer surface. Sci. Rep..

[199] Xue C.H., Ma J.Z. (2013). Long-lived superhydrophobic surfaces. J. Mater. Chem. A..

[200] Yin X.Y., Liu Z.L., Wang D.A., Pei X.W., Yu B., Zhou F. (2015). Bioinspired self-healing organic materials: Chemical mechanisms and fabrications. J. Bionic. Eng..

[201] Li Y., Li L., Sun J.Q. (2010). Bioinspired self-healing superhydrophobic coatings. Angew. Chem. Int. Ed..

[202] Li Y., Chen S.S., Wu M.C., Sun J.Q. (2014). All spraying processes for the fabrication of robust, self-healing, superhydrophobic coatings. Adv. Mater..

[203] Manna U., Lynn D.M. (2013). Restoration of superhydrophobicity in crushed polymer films by treatment with water: Self-healing and recovery of damaged topographic features aided by an unlikely source. Adv. Mater..

[204] Shillingford C., MacCallum N. (2014). Fabrics coated with lubricated nanostructures display robustomniphobicity. Nanotechnology.

[205] Chen S.S., Li X., Li Y., Sun J.Q. (2015). Intumescent flame-retardant and self-healing superhydrophobic coatings on cotton fabric. ACS Nano..

[206] Xue C.H., Zhang Z.D. (2014). Lasting and self-healing superhydrophobic surfaces by coating of polystyrene/SiO_2_ nanoparticles and polydimethylsiloxane. J. Mater. Chem. A..

[207] Si Y.F., Zhu H., Chen L.W., Jinag T., Guo Z.G. (2015). A multifunctional transparent superhydrophobicgel nanocoating with self-healing properties. Chem. Comm..

[208] Chen K.L., Zhou S.X. (2015). Fabrication of all-water-based self-repairing superhydrophbic coatings based on UV-responsive microcapsules. Adv. Funct. Mater..

[209] Esteves A.C.C., Luo Y., van de Put M.W.P. (2014). Self-Replenishing dual structured superhydrophobic coatings prepared by drop-casting of an all-in-one dispersion. Adv. Funct. Mater..

[210] Wu J.X., Li J.Y. (2013). Self-healing of the sueperhydrophobicity by ironing for the abrasion durable superhydrophobic cotton fabrics. Sci. Rep..

[211] Wang H.X., Xue Y.H., Deng J., Feng L.F., Wang X.G., Lin T. (2011). Durable, self-healing superhydrophobic and superoleophobic surfaces from fluorinated-decyl polyhedral oligomeric silsesquioxane and hydrolyzed fluorinated alkyl silane. Angew. Chem. Int. Ed..

[212] Nilghaz A., Wicaksono D.H.B., Gustiono D., Majid F.A.A., Supriyanto E., Kadir M.R.A. (2012). Flexible microfluidic cloth-based analytical devices using a low-cost wax patterning technique. Lab Chip.

[213] Lai Y.K., Pan F., Xu C., Fuchs H., Chi L.F. (2013). *In situ* surface-modification-induced superhydrophobic patterns with reversible wettability and adhesion. Adv. Mater..

[214] Shin B.S., Lee K.R., Moon M.W., Kim H.Y. (2012). Extreme water repellency of nanostructured low-surface-energy non-woven fabrics. Soft Matter.

[215] Rana M., Hao B., Mu L., Chen L., Ma P.C. (2016). Development of multi-functional cotton fabrics with Ag/AgBr-TiO_2_ nanocomposite coating. Compos. Sci. Technol..

[216] Xue C.H., Guo X.J., Zhang M.M., Ma J.Z., Jia S.T. (2015). Fabrication of robust superhydrophobic surfaces by modification of chemically roughened fibers via thiol-ene click chemistry. J. Mater. Chem. A.

[217] Lai Y.K., Lin L.X., Pan F., Huang J.Y., Song R., Huang Y.X., Lin C.J., Fuchs H., Chi L.F. (2013). Bioinspired patterning with extreme wettability contrast on TiO_2_ nanotube array surface: A versatile platform for biomedical applications. Small.

[218] Li H.Q., Lai Y.K., Huang J.Y., Tang Y.X., Yang L., Chen Z., Zhang K.Q., Wang X.C., Tang L.P. (2015). Multifunctional wettability patterns prepared by laser processing on superhydrophobic TiO_2_ nanostructured surfaces. J. Mater. Chem. B.

[219] Lai Y.K., Tang Y.X., Huang J.Y., Pan F., Chen Z., Zhang K.Q., Fuchs H., Chi L.F. (2013). Bioinspired TiO_2_ nanostructure films with special wettability and adhesion for droplets manipulation and patterning. Sci. Rep..

[220] Balu B., Berry A.D., Hess D.W., Breedveld V. (2009). Patterning of superhydrophobic paper to control the mobility of micro-liter drops for two-dimensional lab-on-paper applications. Lab Chip..

[221] Rabnawaz M., Wang Z.J., Wang Y., Wyman L., Hu H., Liu G.J. (2015). Synthesis of poly(dimethylsiloxane)-block-poly[3-(triisopropyloxysilyl) propyl methacrylate] and its use in the facile coating of hydrophilically patterned superhydrophobic fabrics. RSC Adv..

[222] Xing S.Y., Jiang J., Pan T.R. (2013). Interfacial microfluidic transport on micropatterned superhydrophobic textile. Lab Chip.

[223] Huang J.Y., Lai Y.K., Pan F., Wang H., Zhang K.Q., Fuchs H., Chi L.F. (2014). Multifunctional superamphiphobic TiO_2_ nanostructure surfaces with facile wettability and adhesion engineering. Small.

[224] Wang Y., Li X.Y., Hu H., Liu G.J., Rabnawaz M. (2014). Hydrophilically patterned superhydrophobic cotton fabrics and their use in ink printing. J. Mater. Chem. A.

[225] Lai Y., Huang J., Gong J., Huang Y., Wang C., Chen Z., Lin C. (2009). Superhydrophilic-superhydrophobic template: A simple approach to micro- and nanostructure patterning of TiO_2_ films. J. Electrochem. Soc..

[226] Lai Y.K., Lin C.J., Wang H., Huang J.Y., Zhuang H.F., Sun L. (2008). Superhydrophilic-superhydrophobic micropattern on TiO_2_ nanotube films by photocatalytic lithography. Electrochem. Commun..

[227] Zhang Y.Y., Jiang Z.L., Huang J.Y., Lim L.Y., Li W.L., Deng J.Y., Gong D.G., Tang Y.X., Lai Y.K., Chen Z. (2015). Titanate and titania nanostructured materials for environmental and energy applications: A review. RSC Adv..

[228] Su B., Tian Y., Jiang L. (2016). Bioinspired interfaces with superwettability: From materials to chemistry. J. Am. Chem. Soc..

[229] Deng X., Mammen L., Butt H.J., Vollmer D. (2012). Candle soot as a template for a transparent robust superamphiphobic coating. Science.

[230] Tuteja A., Choi W., Ma M.L., Mabry J.M., Mazzella S.A., Rutledge G.C., McKinley G.H., Cohen R.E. (2007). Designing superoleophobic surfaces. Science.

[231] Lu Y., Sathasivam S., Song J.L., Crick C.R., Carmalt C.J., Parkin I.P. (2015). Robust self-cleaning surfaces that function when exposed to either air or oil. Science.

